# Supplementary material for: Synthesis, Structural Characterization, and Biological Activity of New Pyrazolo[4,3-e][1,2,4]triazine Acyclonucleosides
Source: Molecules. 2020 Jan 5;25(1):221. doi: 10.3390/molecules25010221 (PMC6982861; doi:10.3390/molecules25010221)
Supplement: Supplementary file 1 [file molecules-25-00221-s001.pdf]

SUPPLEMENTARY MATERIALS

Synthesis, structural characterization and biological activity of new pyrazolo[4,3-*e*][1,2,4]triazine acyclonucleosides

Mariusz Mojzych[a*], Zofia Bernat[a], Zbigniew Karczmarzyk[a], Joanna Matysiak[b], Andrzej Fruziński[c]

[a] *Siedlce University of Natural Sciences and Humanities, Department of Chemistry, ul. 3 Maja 54, 08-110 Siedlce, Poland. e-mail: mmojzych@yahoo.com*

[b] *University of Life Sciences, Department of Chemistry, Akademicka 15, 20-950, Lublin, Poland*

[c]*Technical University, Department of General and Ecological Chemistry, ul. Żeromskiego 115, 90-924 Łódź, Poland*

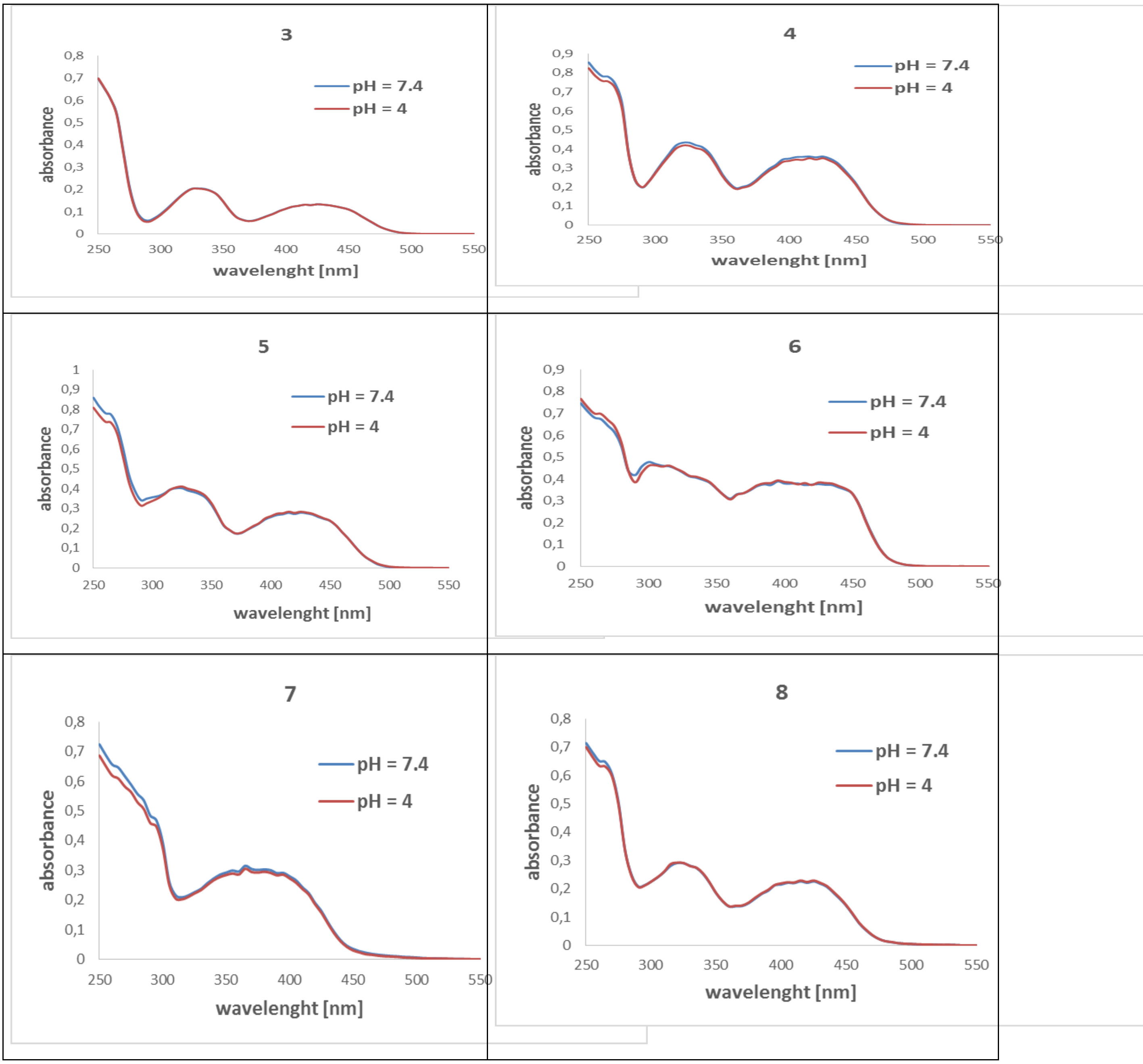

Fig. 1S. UV–Vis spectra of compounds **3-8** in the aqueous methanol solution of different pH

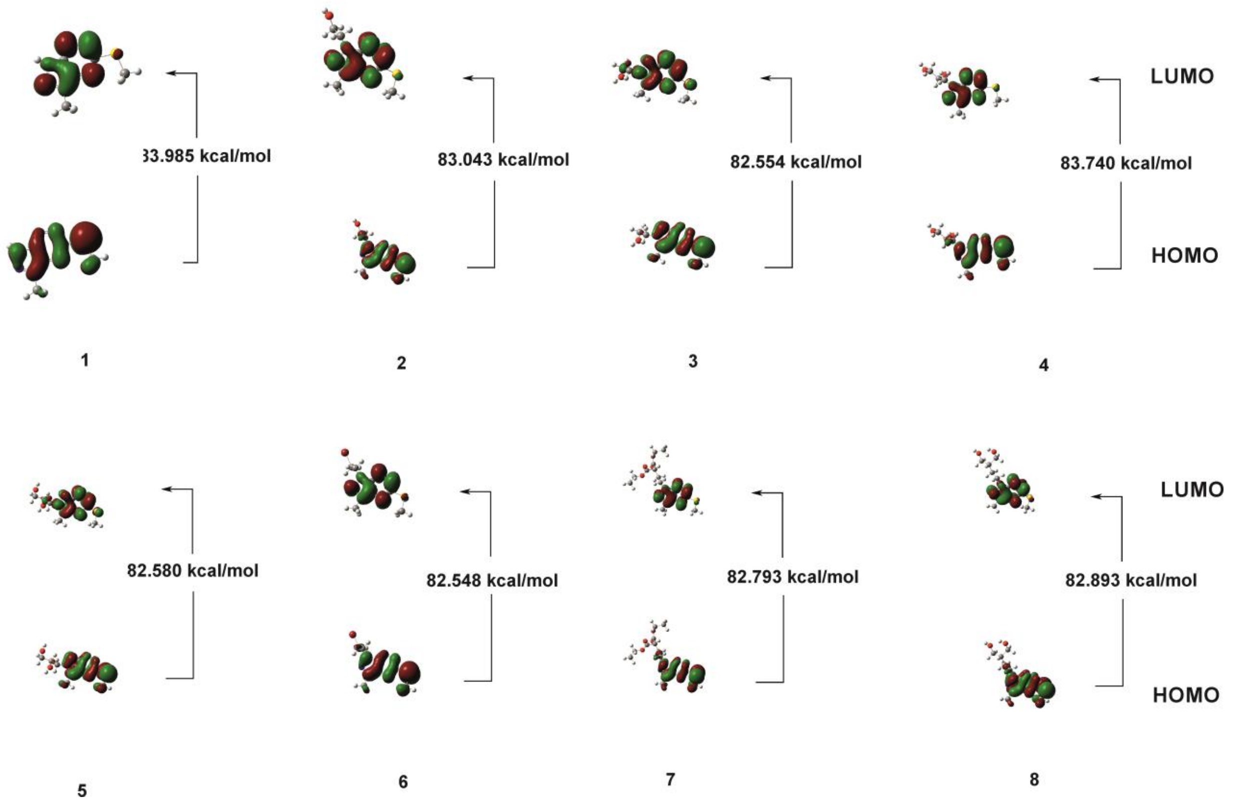

Fig. 2S. Schematic representation of the HOMO and LUMO orbitals of **1**–**8** as calculated at DFT/B3LYP/6-311++G(d,p) level.

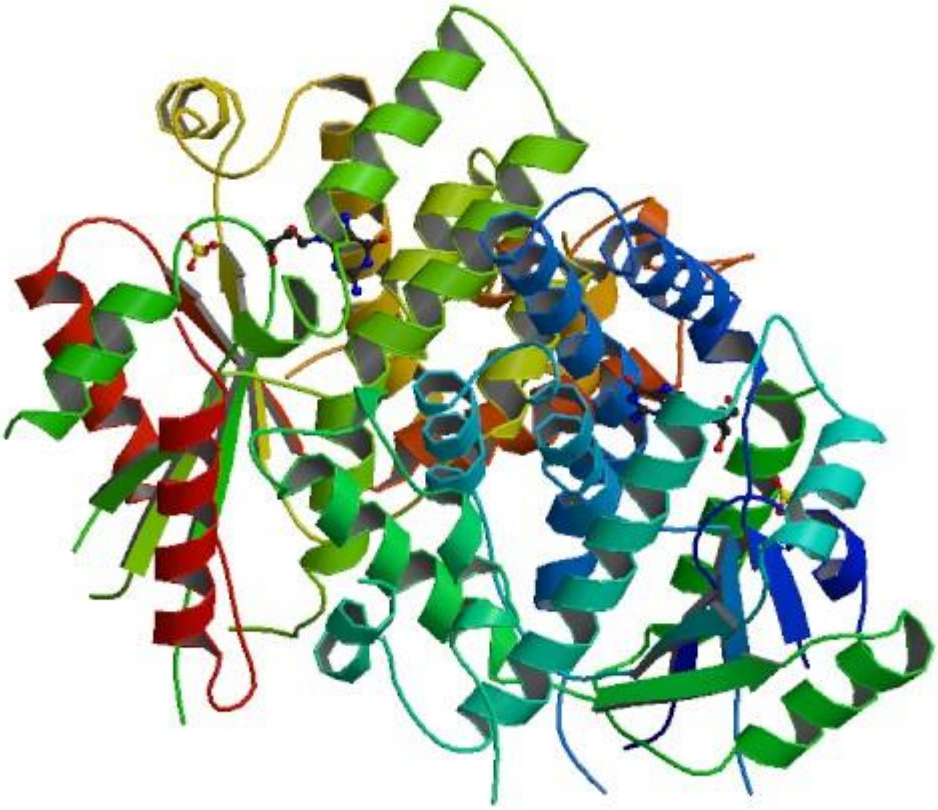

Fig. 3S. The crystal structure of TK enzyme.

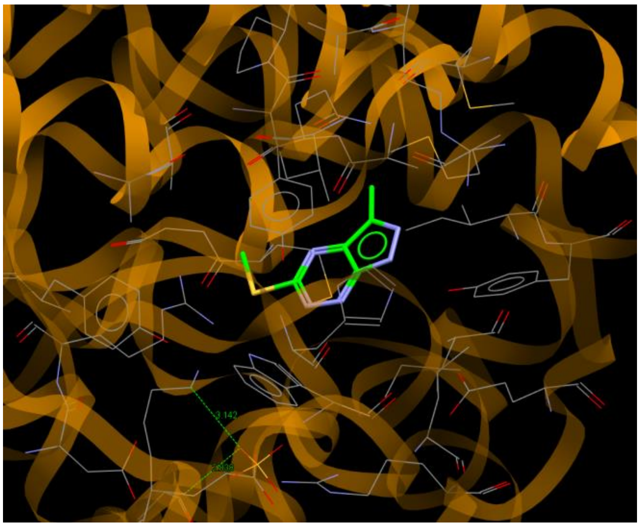

1

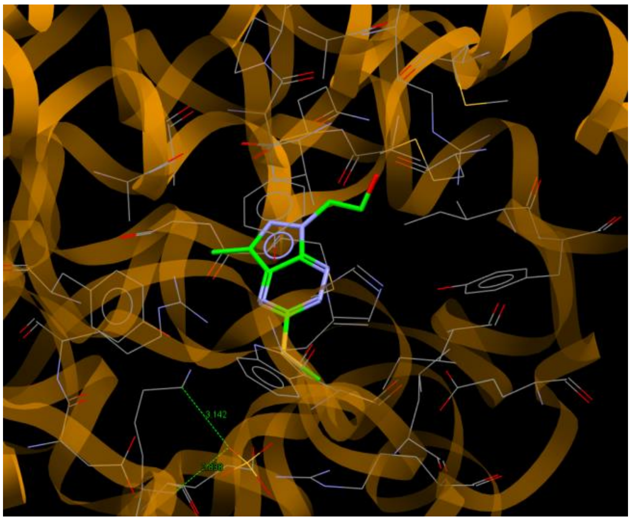

2

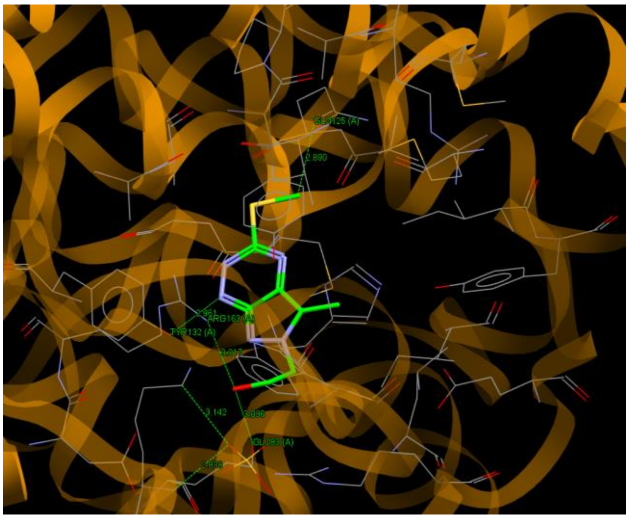

3

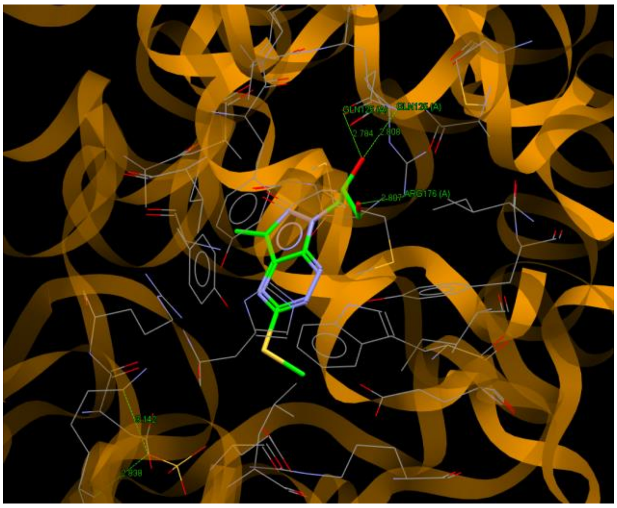

4

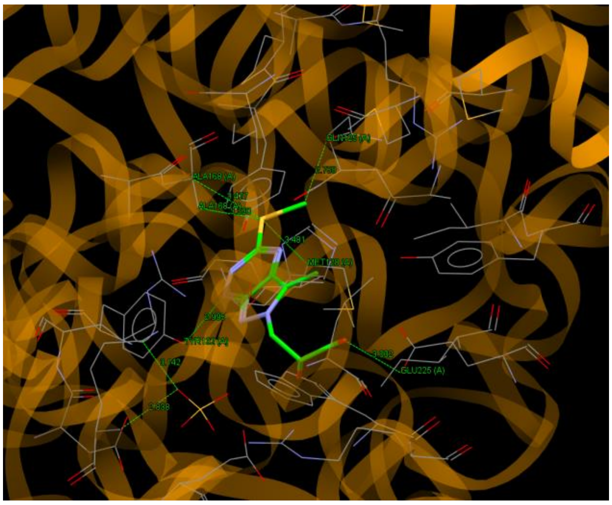

5

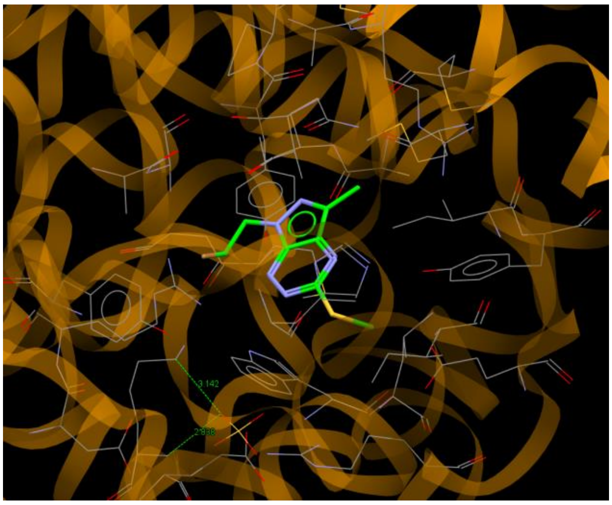

6

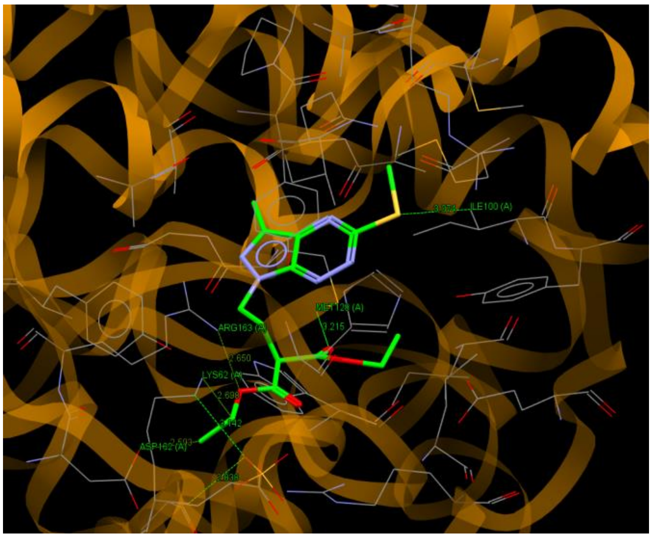

7

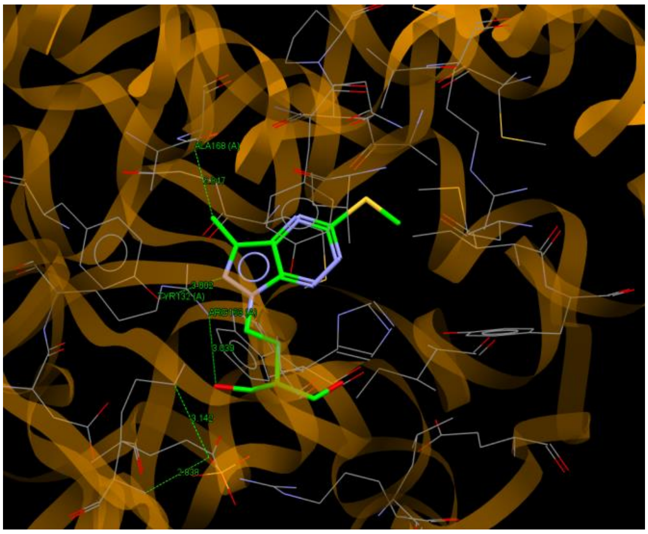

8

Fig 4S. A view of the interaction of **1**–**8** and with amino acids of binding site in TK.

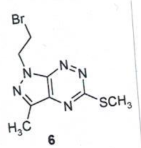

6

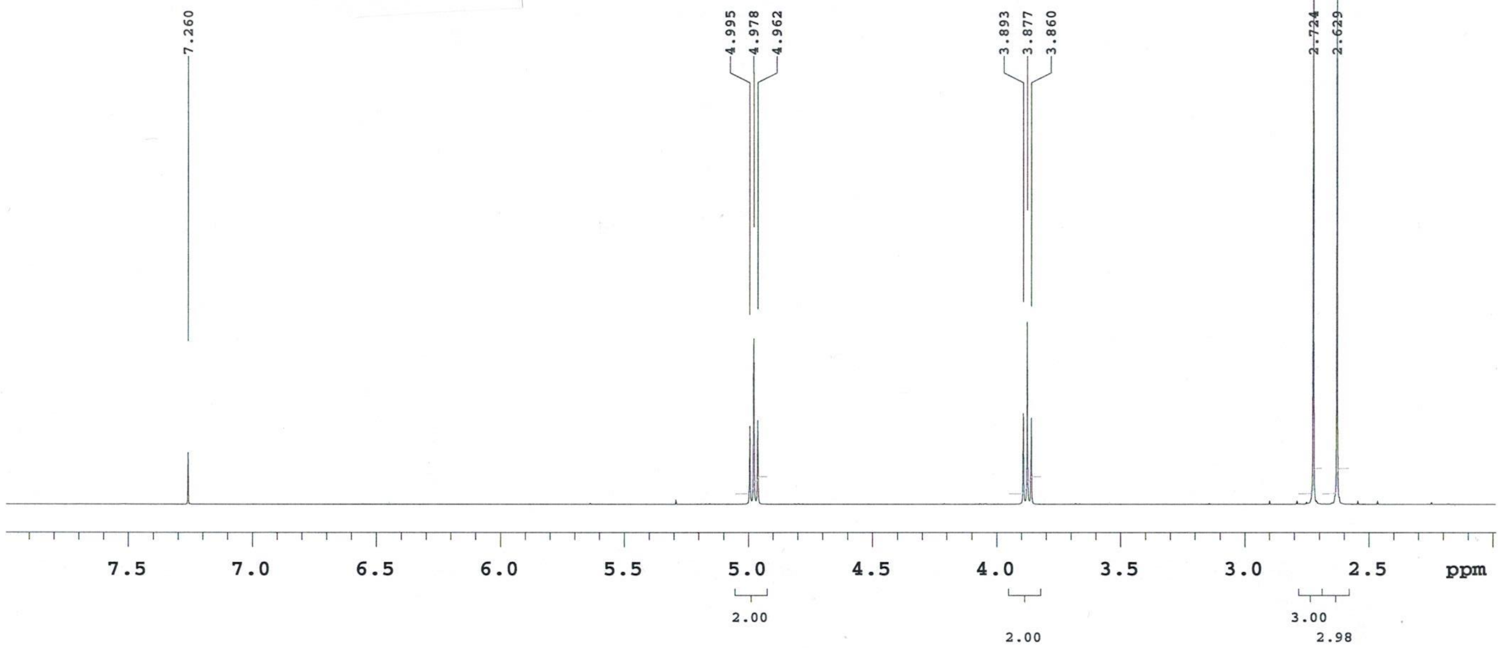

PULSE SEQUENCE
Relax. delay 1.000 sec
Pulse 45.0 degrees
Acq. time 4.797 sec
Width 6830.6 Hz
48 repetitions

OBSERVE H1, 399.6978692

DATA PROCESSING
FT size 65536
Total time 4 minutes

Sample Name:
MM638_N1
Data Collected on:
400MR-vnmrs400
Archive directory:
[illegible]

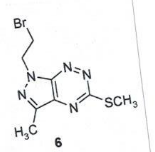

6

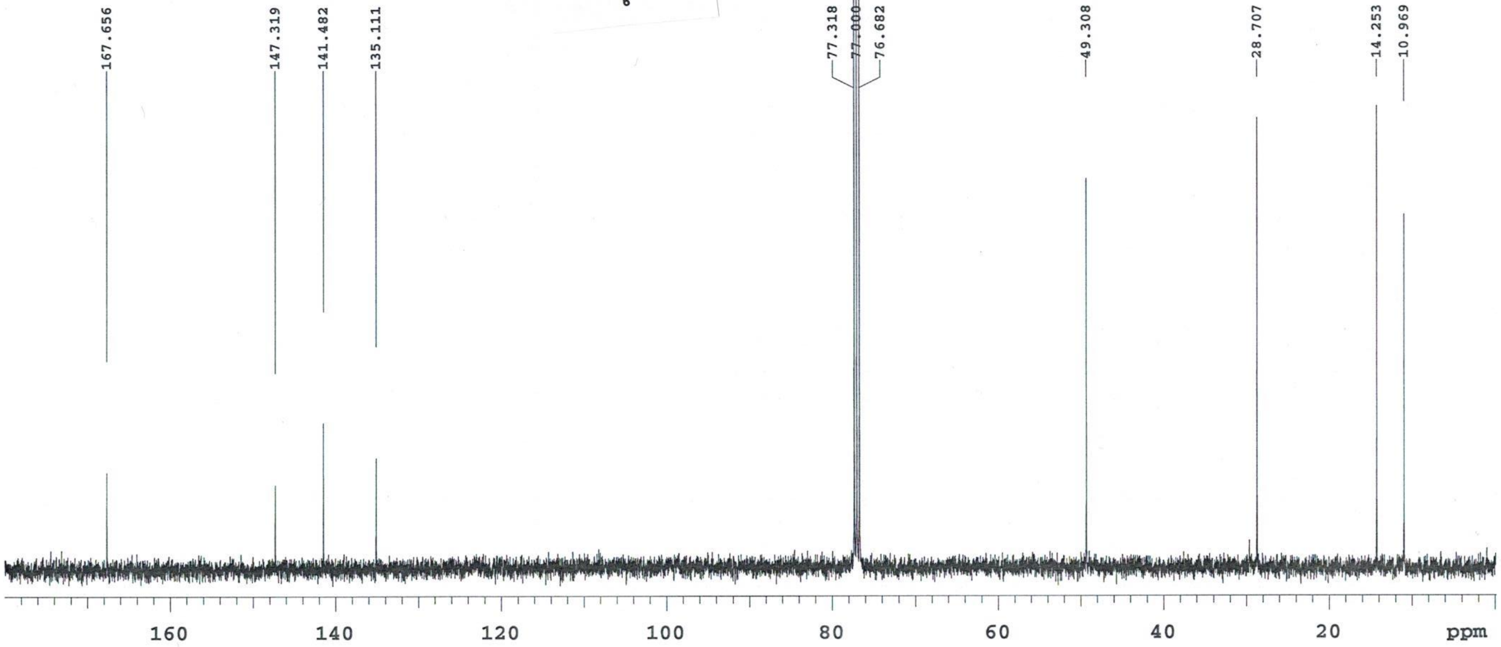

PULSE SEQUENCE
Relax. delay 2.000 sec
Pulse 45.0 degrees
Acq. time 2.000 sec
Width 25510.2 Hz
768 repetitions

OBSERVE C13, 100.5041103
DECOUPLE H1, 399.6998746
Power 34 dB
continuously on
WALTZ-16 modulated

DATA PROCESSING
Line broadening 1.0 Hz
FT size 131072
Total time 51 minutes

Sample Name:
MM638N-1
Data Collected on:
400MR-vnmrs400
Archive directory:
[illegible]

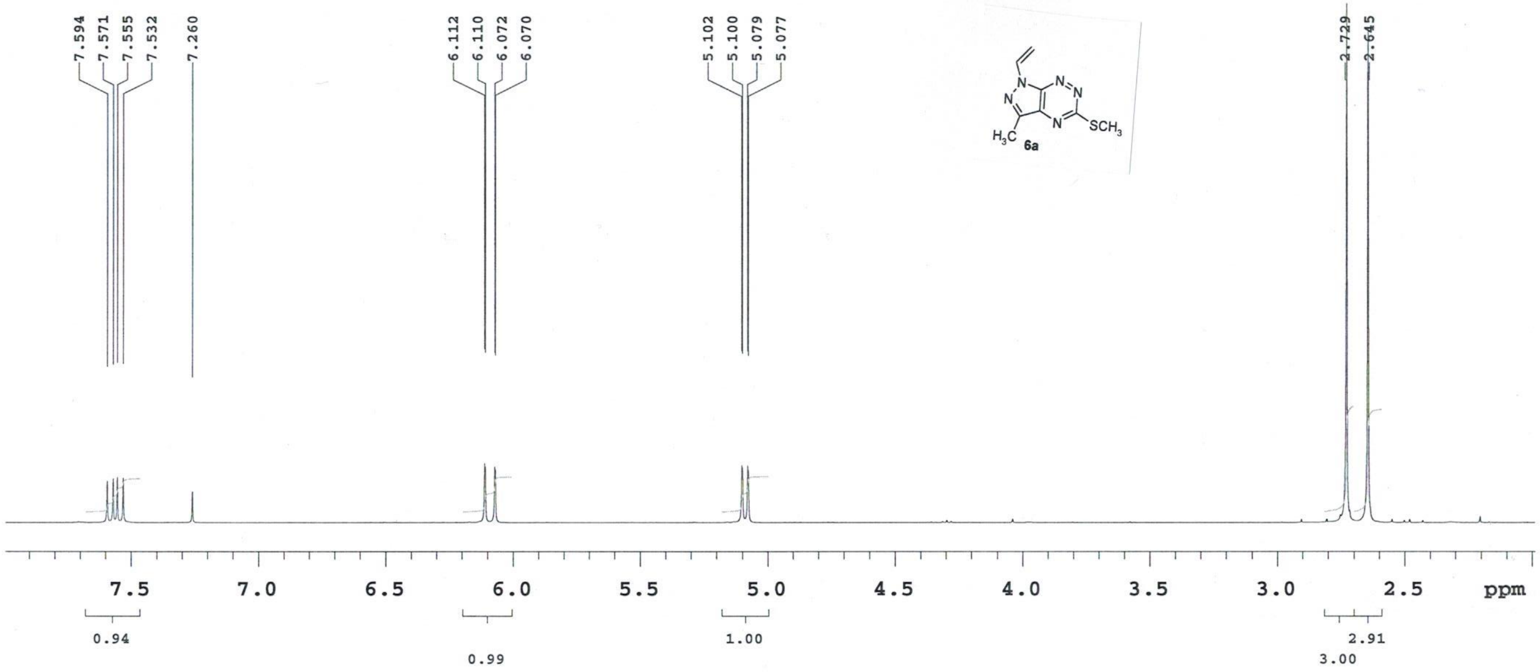

PULSE SEQUENCE
Relax. delay 1.000 sec
Pulse 45.0 degrees
Acq. time 4.797 sec
Width 6830.6 Hz
32 repetitions

OBSERVE H1, 399.6978692

DATA PROCESSING
FT size 65536
Total time 3 minutes

Sample Name:
MM636F1
Data Collected on:
400MR-vnmrs400
Archive directory:
[illegible]

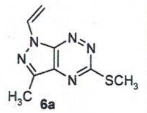

6a

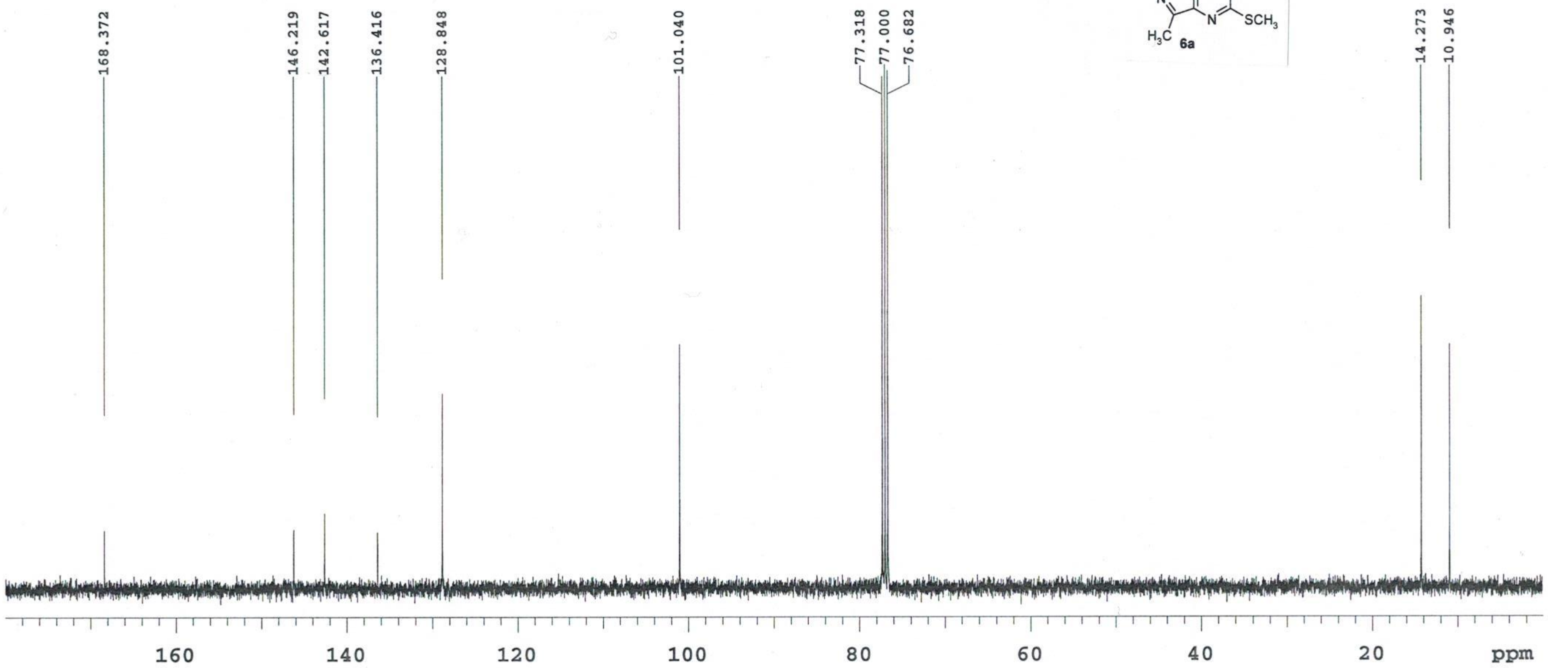

PULSE SEQUENCE
Relax. delay 2.000 sec
Pulse 45.0 degrees
Acq. time 2.000 sec
Width 25510.2 Hz
384 repetitions

OBSERVE C13, 100.5041099
DECOUPLE H1, 399.6998746
Power 34 dB
continuously on
WALTZ-16 modulated

DATA PROCESSING
Line broadening 1.0 Hz
FT size 131072
Total time 25 minutes

Sample Name:
MM636F1
Data Collected on:
400MR-vnmrs400
Archive directory:
[illegible]

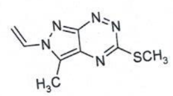

6b

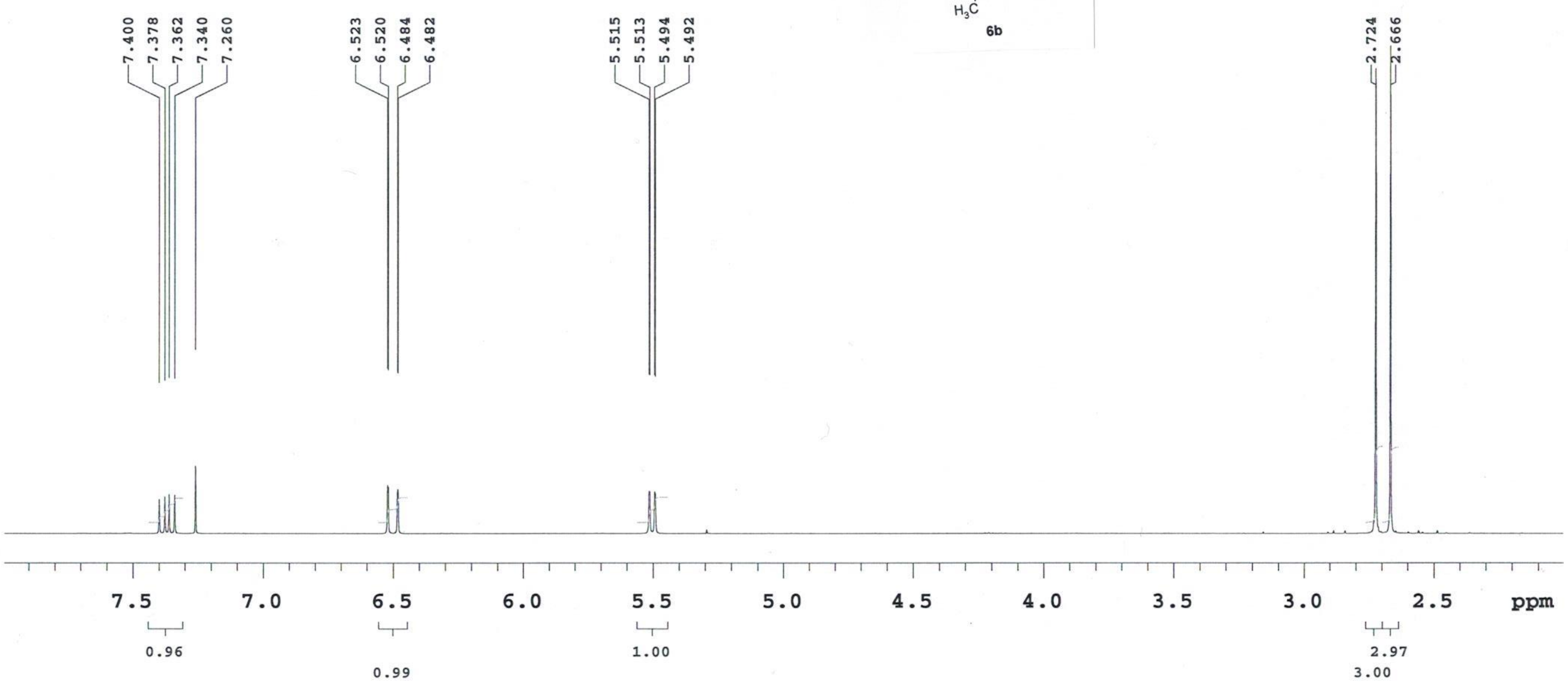

PULSE SEQUENCE
Relax. delay 1.000 sec
Pulse 45.0 degrees
Acq. time 4.797 sec
Width 6830.6 Hz
40 repetitions

OBSERVE H1, 399.6978692

DATA PROCESSING
FT size 65536
Total time 3 minutes

Sample Name:
mm638n-2
Data Collected on:
400MR-vnmrs400
Archive directory:
[illegible]

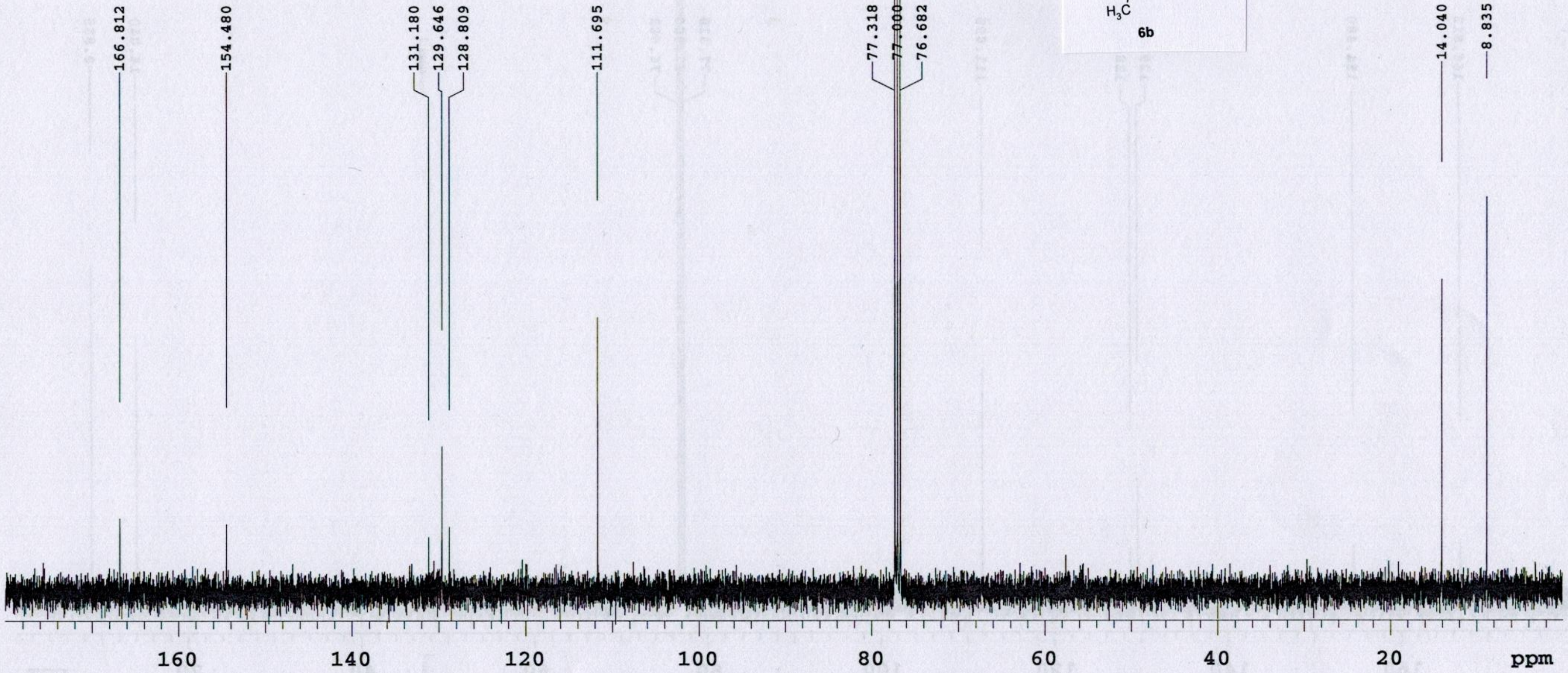

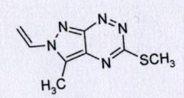

6b

PULSE SEQUENCE
Relax. delay 2.000 sec
Pulse 45.0 degrees
Acq. time 2.000 sec
Width 25510.2 Hz
512 repetitions

OBSERVE C13, 100.5041095
DECOUPLE H1, 399.6998746
Power 34 dB
continuously on
WALTZ-16 modulated

DATA PROCESSING
Line broadening 0.5 Hz
FT size 131072
Total time 34 minutes

Sample Name:
MM638N-2
Data Collected on:
400MR-vnmrs400
Archive directory:
[illegible]

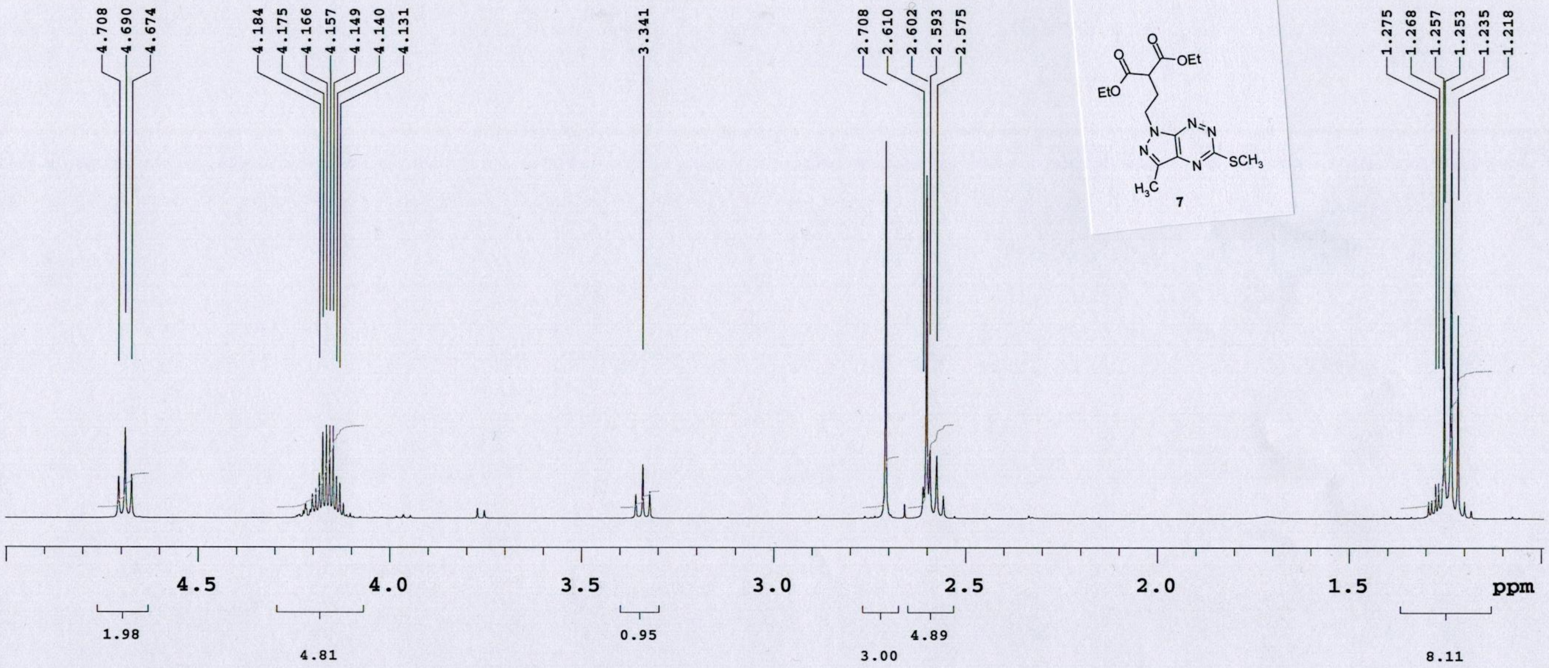

PULSE SEQUENCE
Relax. delay 1.000 sec
Pulse 45.0 degrees
Acq. time 4.797 sec
Width 6830.6 Hz
128 repetitions

OBSERVE H1, 399.6978690

DATA PROCESSING
FT size 65536
Total time 12 minutes

MM640 w CDCl3

Sample Name:
MM640
Data Collected on:
400MR-vnmrs400
Archive directory:
[illegible]

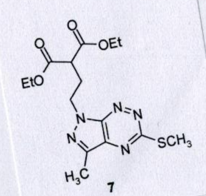

7

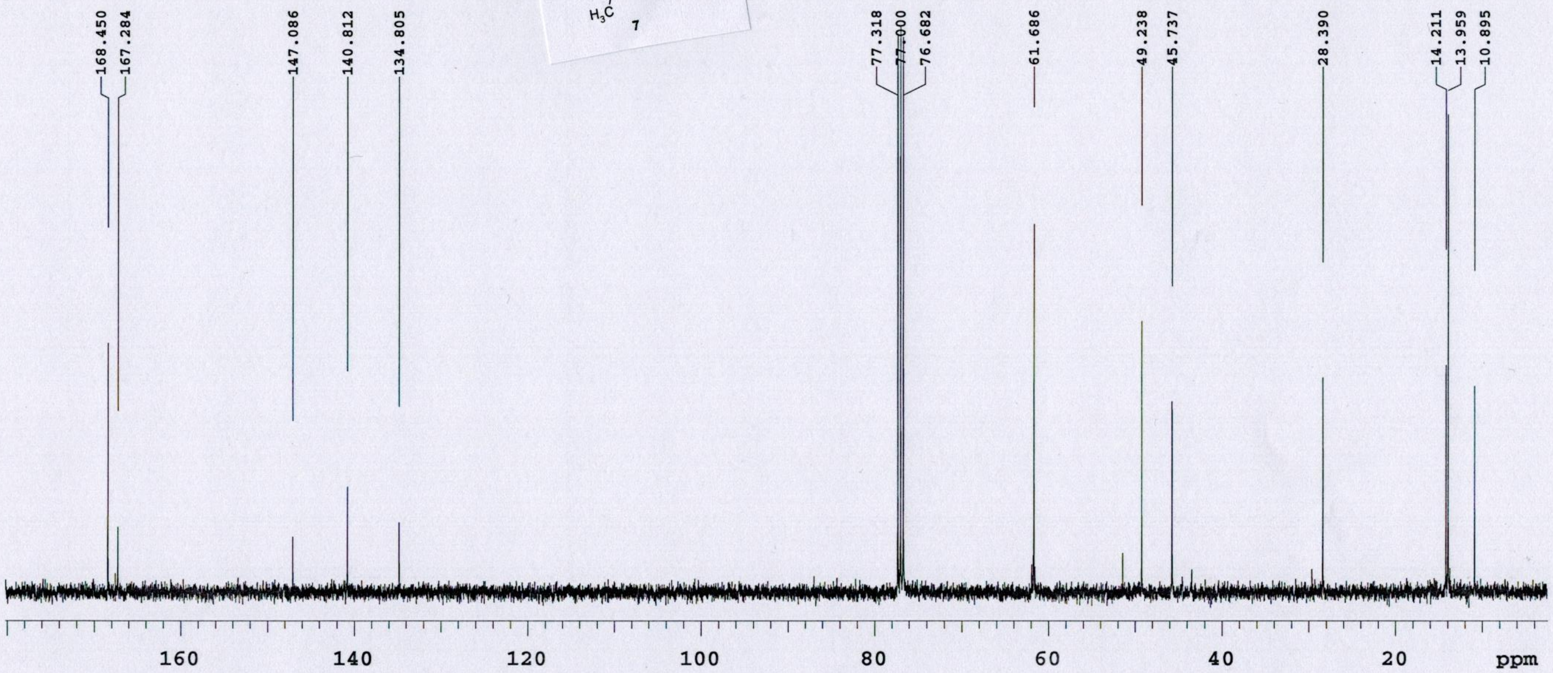

PULSE SEQUENCE
Relax. delay 2.000 sec
Pulse 45.0 degrees
Acq. time 2.000 sec
Width 25510.2 Hz
352 repetitions

OBSERVE C13, 100.5041106
DECOUPLE H1, 399.6998746
Power 34 dB
continuously on
WALTZ-16 modulated

DATA PROCESSING
Line broadening 1.0 Hz
FT size 131072
Total time 23 minutes

lank1 w CDCl3

Sample Name:
lank1
Data Collected on:
400MR-vnmrs400
Archive directory:
[illegible]

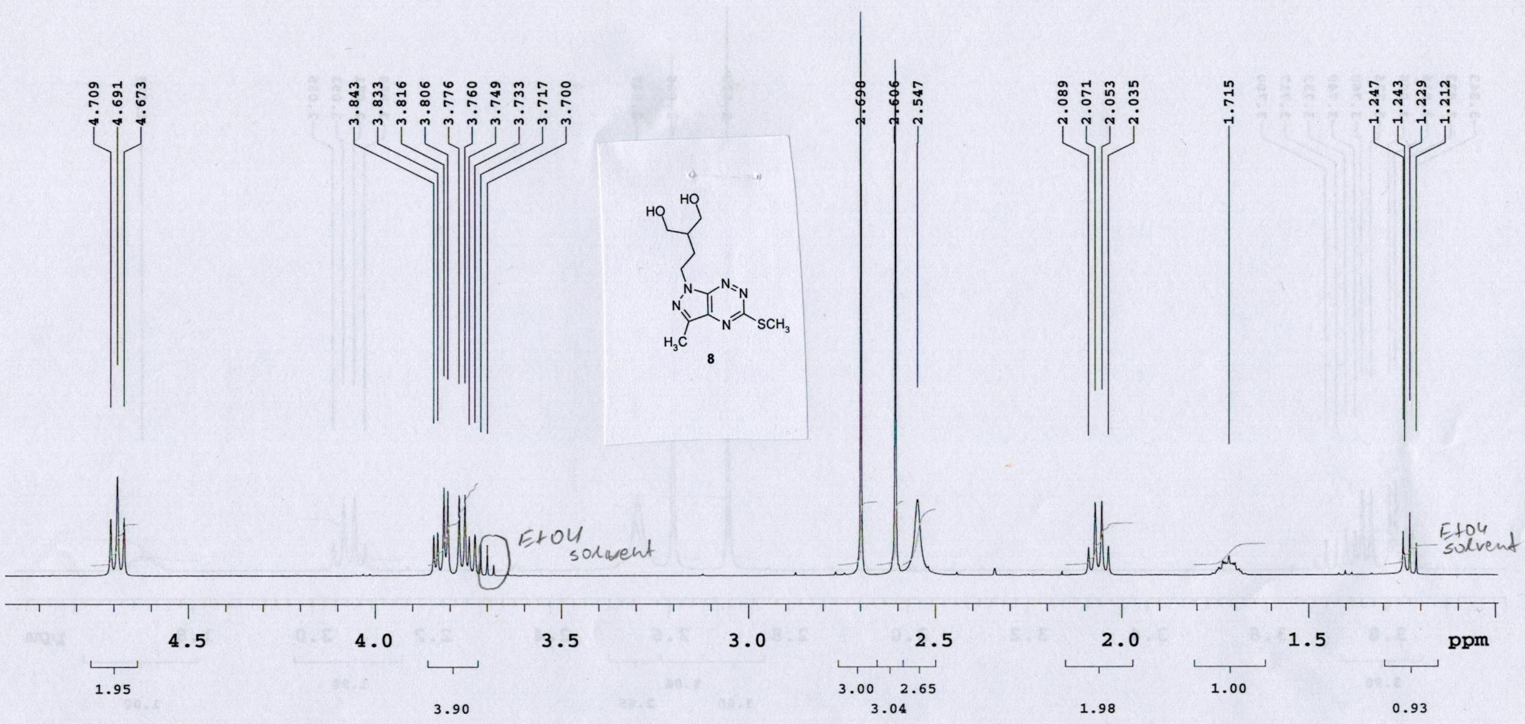

PULSE SEQUENCE
Relax. delay 1.000 sec
Pulse 42.6 degrees
Acq. time 4.797 sec
Width 6830.6 Hz
164 repetitions

OBSERVE H1, 399.6978692

DATA PROCESSING
FT size 65536
Total time 15 minutes

MM641F2 w CDCl3

Sample Name:
MM641F2
Data Collected on:
400MR-vnmrs400
Archive directory:

[illegible]

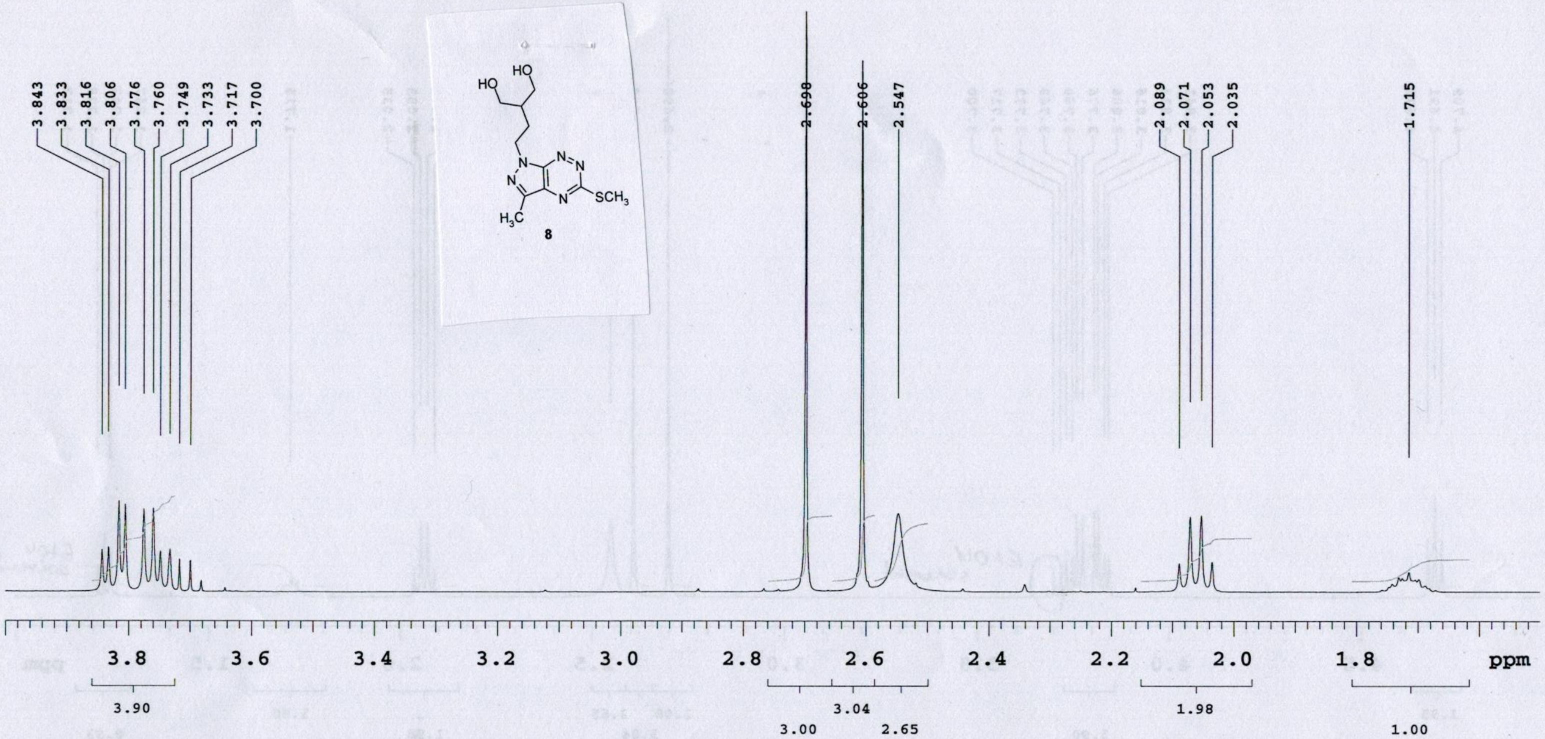

PULSE SEQUENCE
Relax. delay 1.000 sec
Pulse 42.6 degrees
Acq. time 4.797 sec
Width 6830.6 Hz
164 repetitions

OBSERVE H1, 399.6978692

DATA PROCESSING
FT size 65536
Total time 15 minutes

MM641F2 w CDCl3

Sample Name:
MM641F2
Data Collected on:
400MR-vnmrs400
Archive directory:
[illegible]

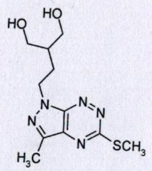

8

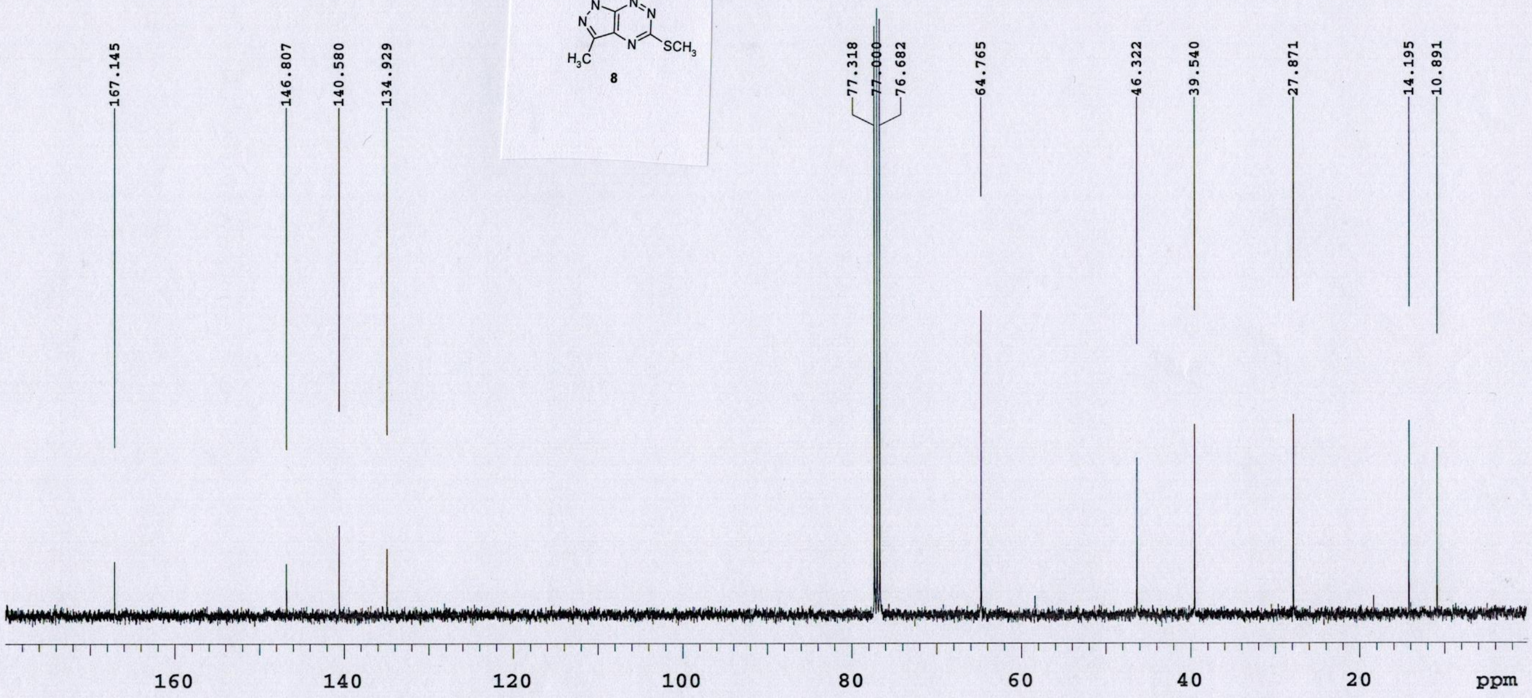

PULSE SEQUENCE
Relax. delay 2.000 sec
Pulse 45.0 degrees
Acq. time 2.000 sec
Width 25510.2 Hz
704 repetitions

OBSERVE C13, 100.5041103
DECOUPLE H1, 399.6998746
Power 34 dB
continuously on
WALTZ-16 modulated

DATA PROCESSING
Line broadening 1.0 Hz
FT size 131072
Total time 46 minutes

MM641F2 w CDCl3

Sample Name:
MM641F2
Data Collected on:
400MR-vnmrs400
Archive directory:
[illegible]

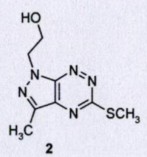

2

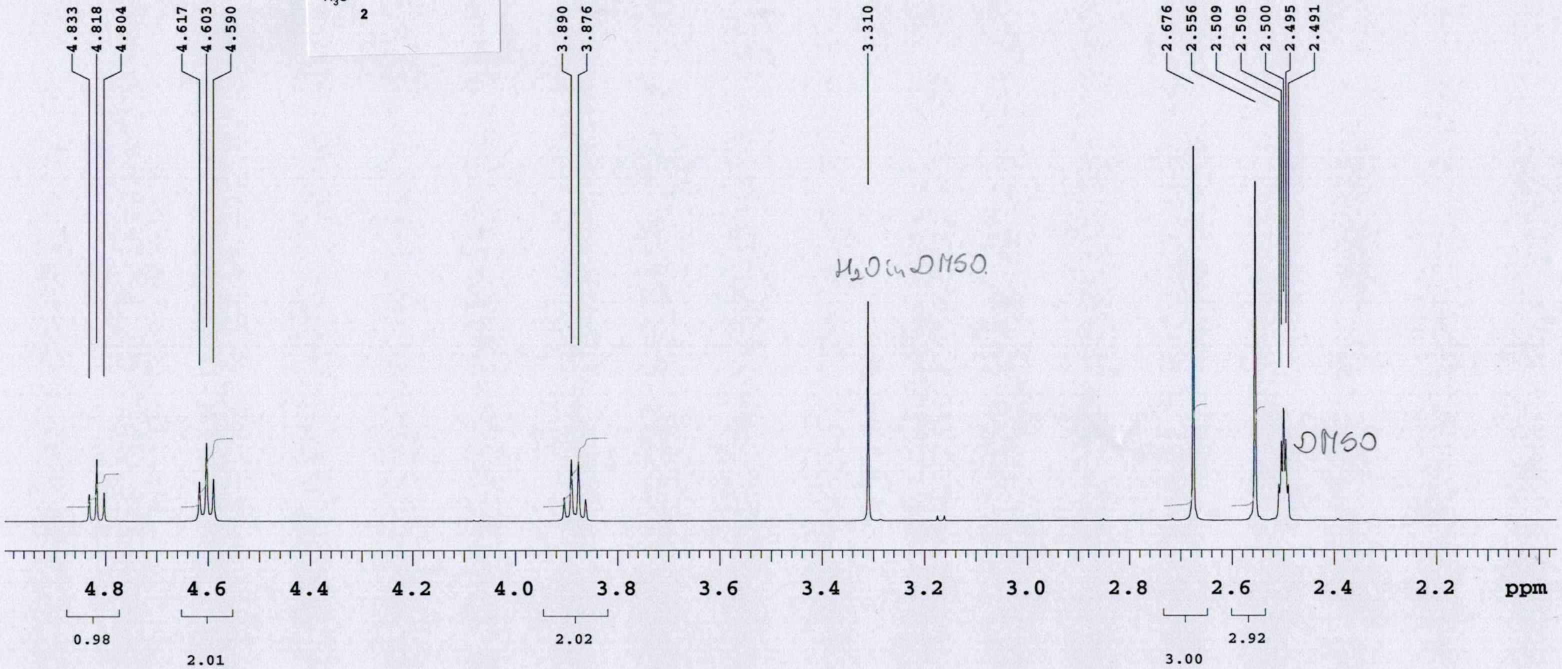

PULSE SEQUENCE
Relax. delay 1.000 sec
Pulse 45.0 degrees
Acq. time 4.797 sec
Width 6830.6 Hz
28 repetitions

OBSERVE H1, 399.6902569

DATA PROCESSING
FT size 65536
Total time 2 minutes

Sample Name:
MMKG21F1
Data Collected on:
400MR-vnmrs400
Archive directory:
[illegible]

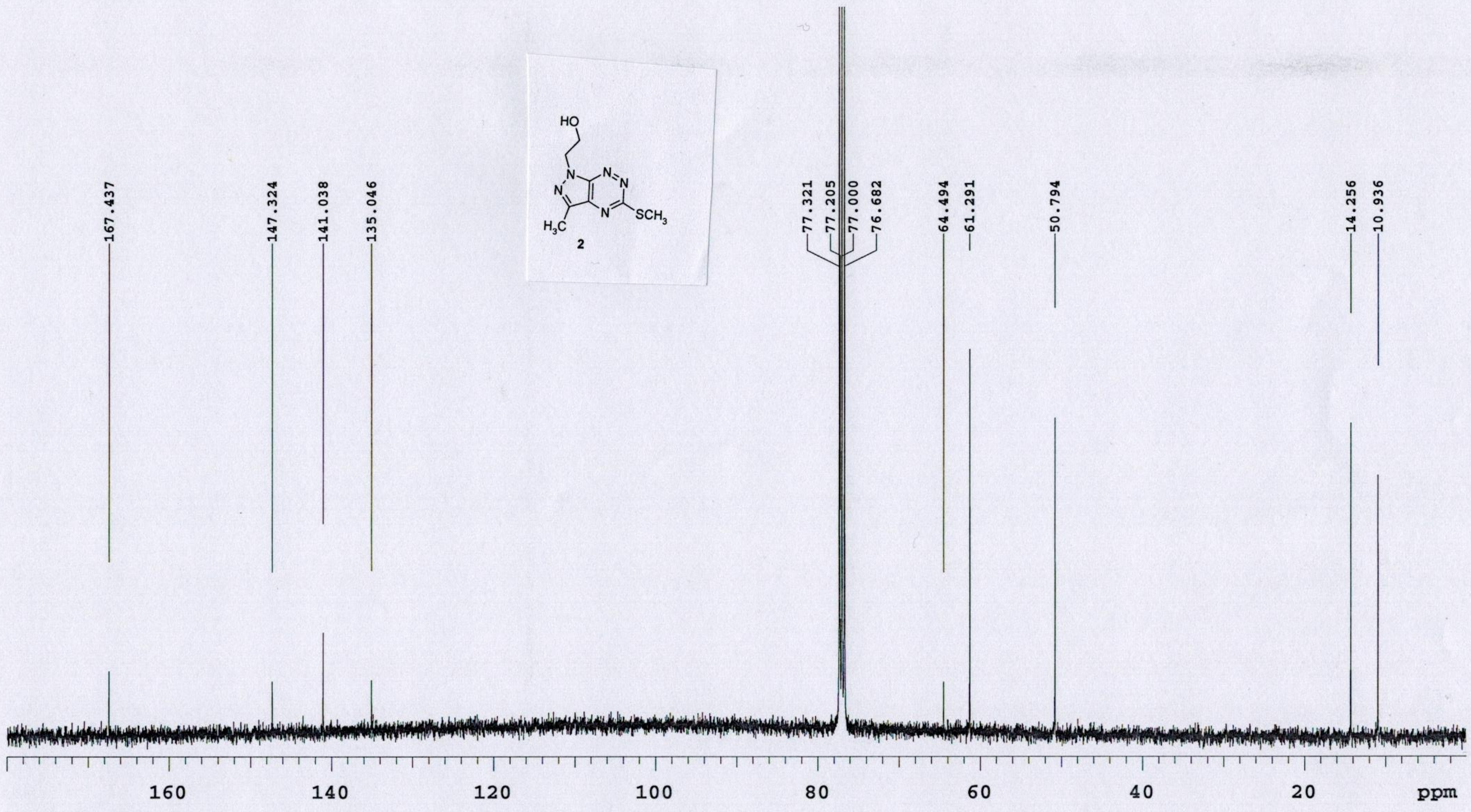

PULSE SEQUENCE
Relax. delay 2.000 sec
Pulse 45.0 degrees
Acq. time 2.000 sec
Width 25510.2 Hz
10000 repetitions

OBSERVE C13, 100.5017186
DECOUPLE H1, 399.6903662
Power 34 dB
continuously on
WALTZ-16 modulated

DATA PROCESSING
Line broadening 1.0 Hz
FT size 131072
Total time 11.1 hours

mm653f1 w cdcl3

Sample Name:
mm653f1
Data Collected on:
400MR-vnmrs400
Archive directory:
[illegible]

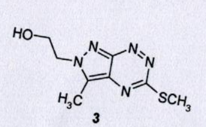

3

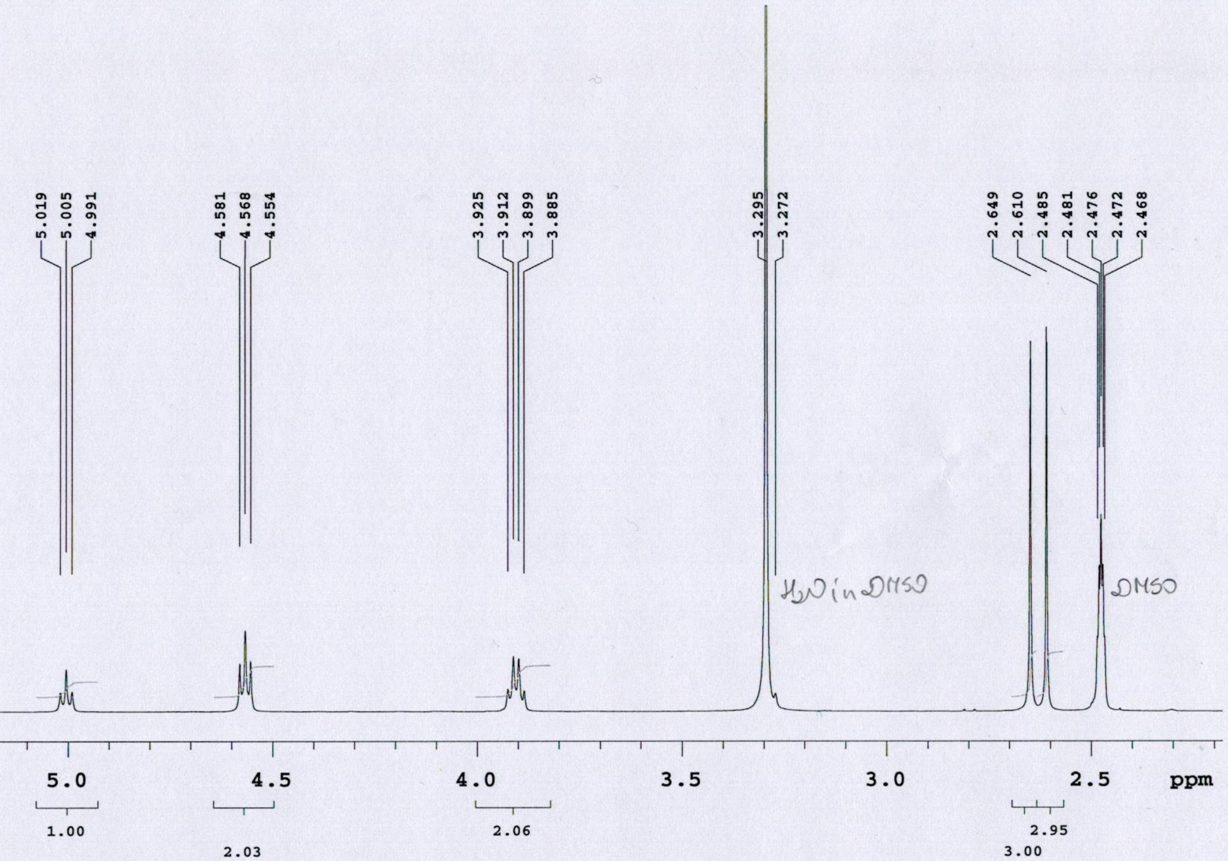

PULSE SEQUENCE
Relax. delay 1.000 sec
Pulse 45.0 degrees
Acq. time 4.797 sec
Width 6830.6 Hz
52 repetitions

OBSERVE H1, 399.6902663

DATA PROCESSING
FT size 65536
Total time 5 minutes

Sample Name:
MMKG21F2
Data Collected on:
400MR-vnmrs400
Archive directory:
[illegible]

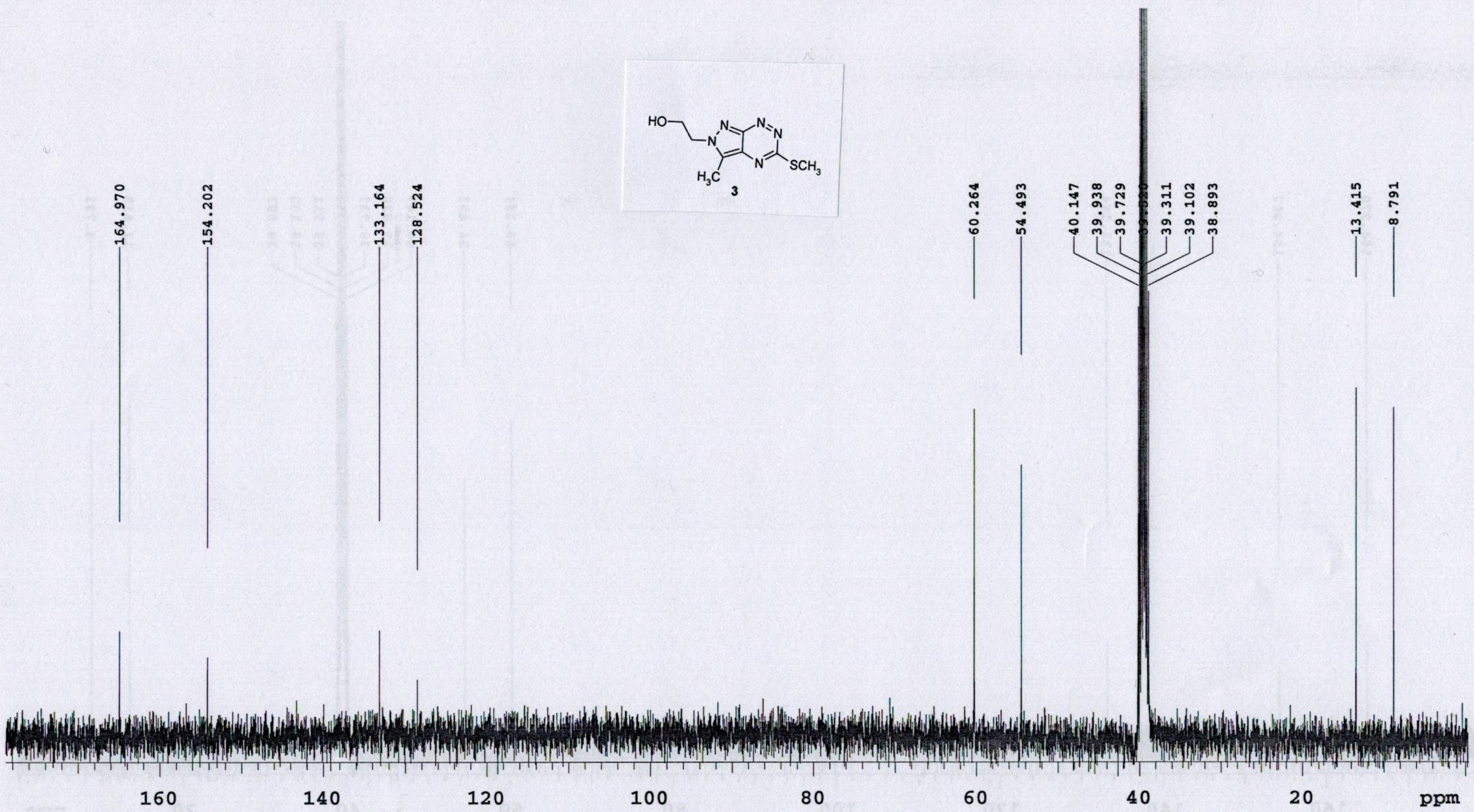

PULSE SEQUENCE
Relax. delay 1.500 sec
Pulse 45.0 degrees
Acq. time 1.285 sec
Width 25510.2 Hz
1664 repetitions

OBSERVE C13, 100.5022431
DECOUPLE H1, 399.6922647
Power 34 dB
continuously on
WALTZ-16 modulated

DATA PROCESSING
Line broadening 1.0 Hz
FT size 65536
Total time 77 minutes

Sample Name:
MMKG21F2
Data Collected on:
400MR-vnmrs400
Archive directory:
[illegible]

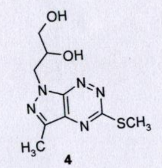

4

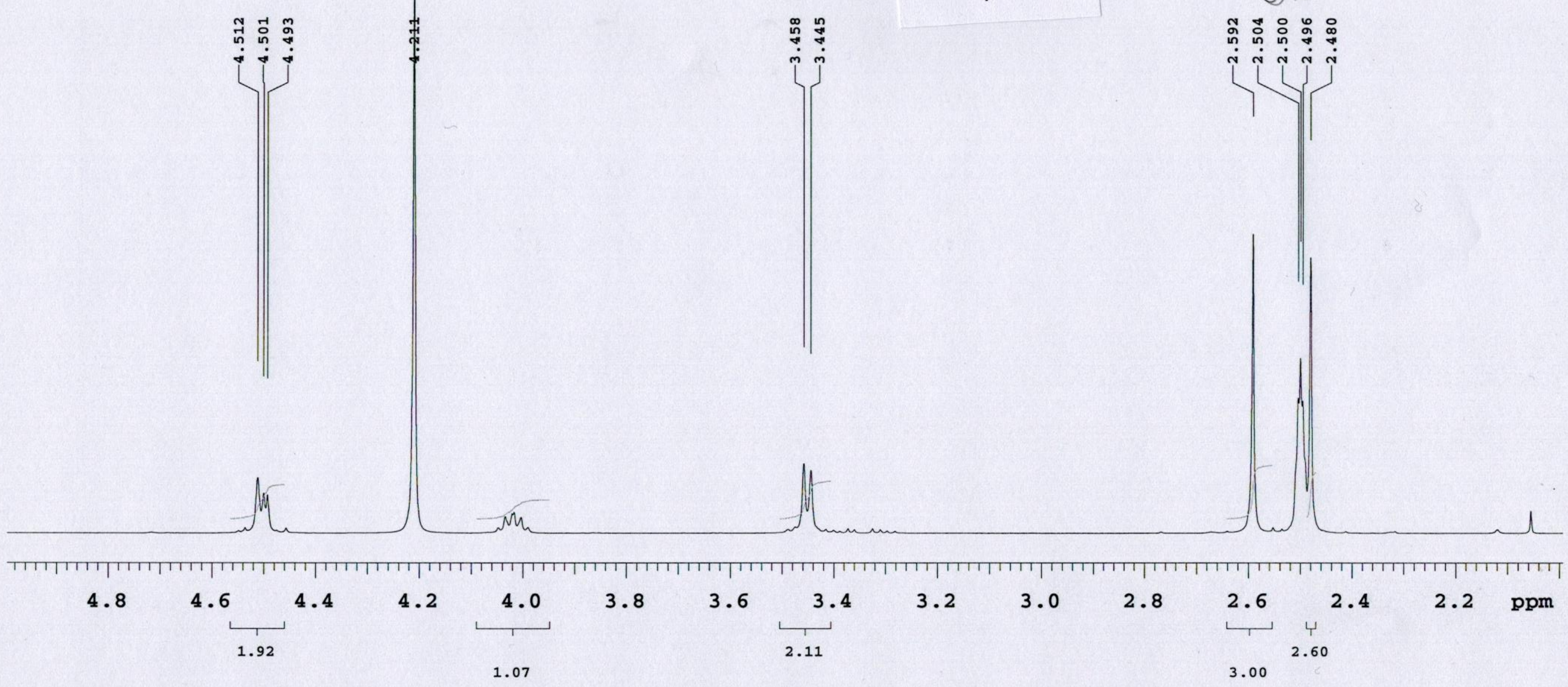

PULSE SEQUENCE
Relax. delay 1.000 sec
Pulse 45.0 degrees
Acq. time 4.797 sec
Width 6830.6 Hz
68 repetitions

OBSERVE H1, 399.6902577

DATA PROCESSING
FT size 65536
Total time 6 minutes

Sample Name:
MMKG22F1
Data Collected on:
400MR-vnmrs400
[illegible]

VARIAN

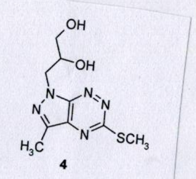

4

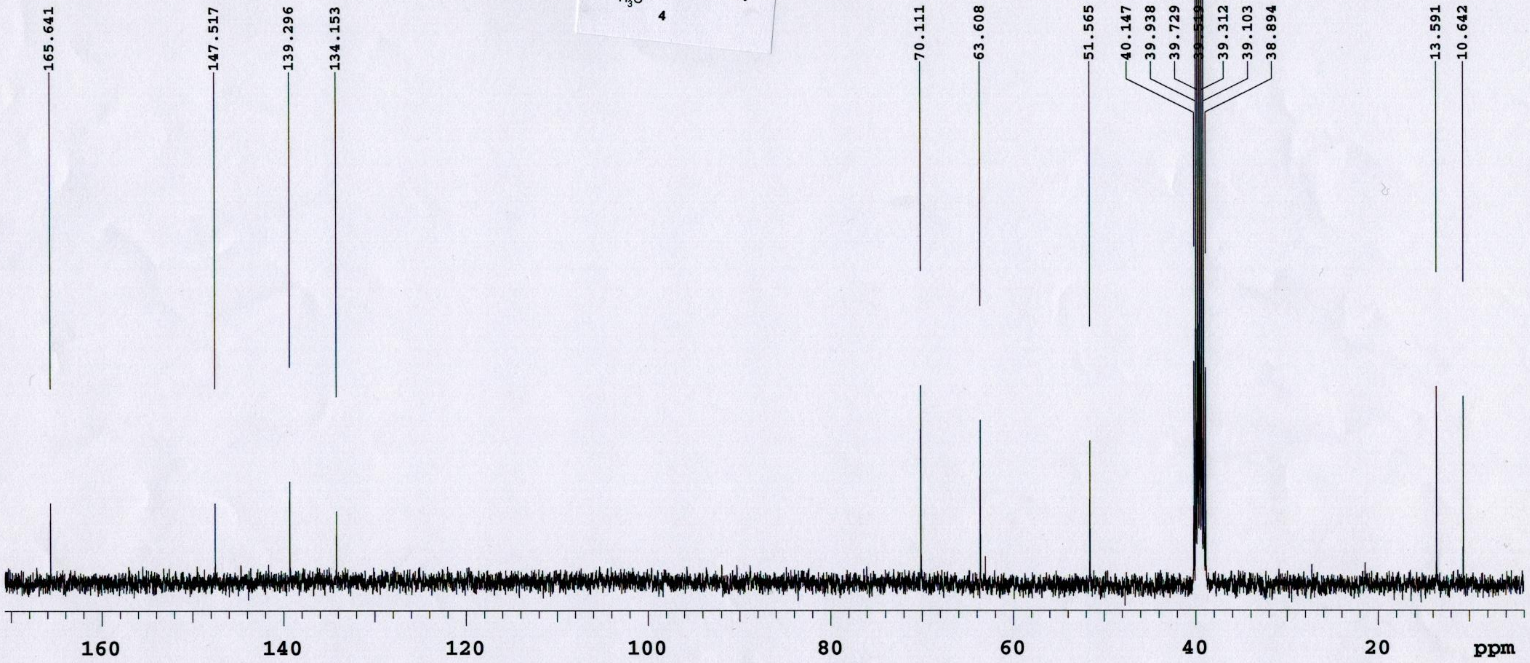

PULSE SEQUENCE
Relax. delay 2.000 sec
Pulse 45.0 degrees
Acq. time 2.000 sec
Width 25510.2 Hz
736 repetitions

OBSERVE C13, 100.5022438
DECOUPLE H1, 399.6922647
Power 34 dB
continuously on
WALTZ-16 modulated

DATA PROCESSING
Line broadening 1.0 Hz
Gauss apodization 0.600 sec
FT size 262144
Total time 49 minutes

Sample Name:
MMKG22F1
Data Collected on:
400MR-vnmrs400
Archive directory:
[illegible]

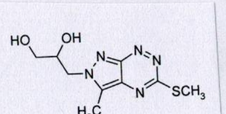

5

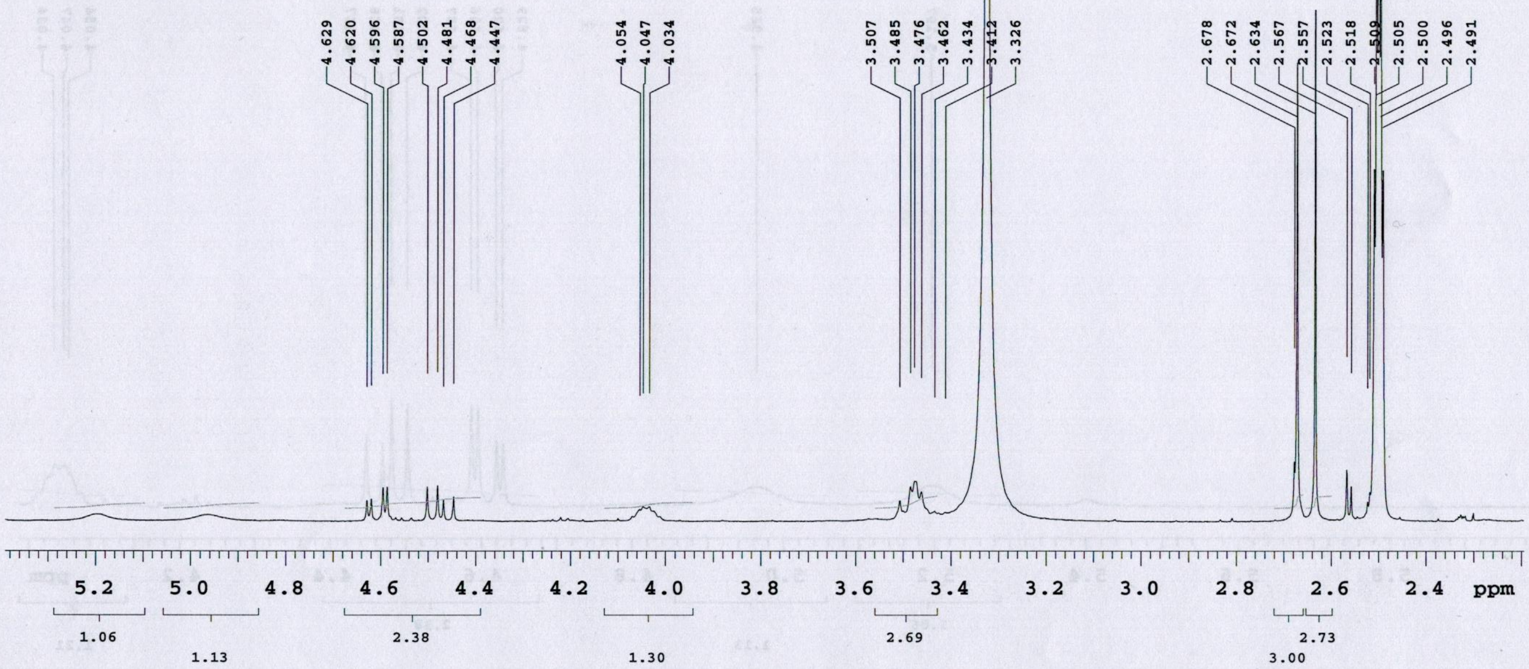

PULSE SEQUENCE
Relax. delay 1.000 sec
Pulse 45.0 degrees
Acq. time 4.797 sec
Width 6830.6 Hz
108 repetitions

OBSERVE H1, 399.6902566

DATA PROCESSING
FT size 65536
Total time 10 minutes

Sample Name:
MMKG22F2
Data Collected on:
400MR-vnmrs400
Archive directory: [illegible]

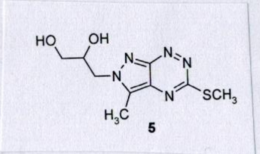

5

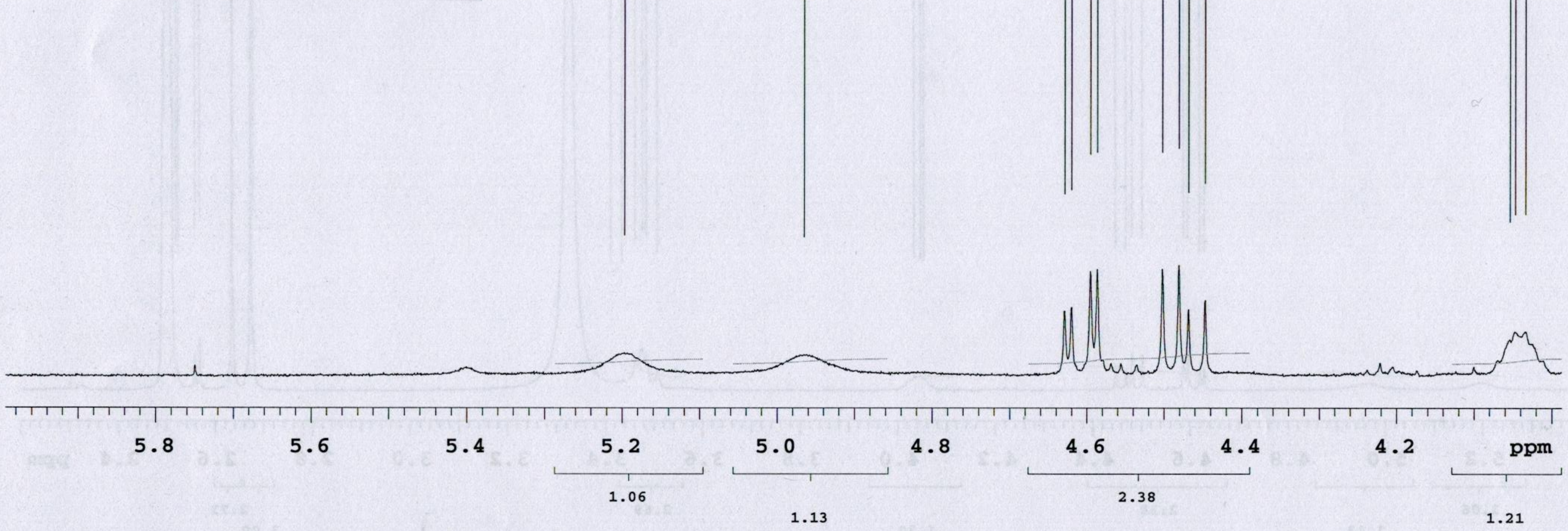

PULSE SEQUENCE
Relax. delay 1.000 sec
Pulse 45.0 degrees
Acq. time 4.797 sec
Width 6830.6 Hz
108 repetitions

OBSERVE H1, 399.6902566

DATA PROCESSING
FT size 65536
Total time 10 minutes

Sample Name:
MMKG22F2
Data Collected on:
400MR-vnmrs400
Archive directory:
[illegible]

Compound 1

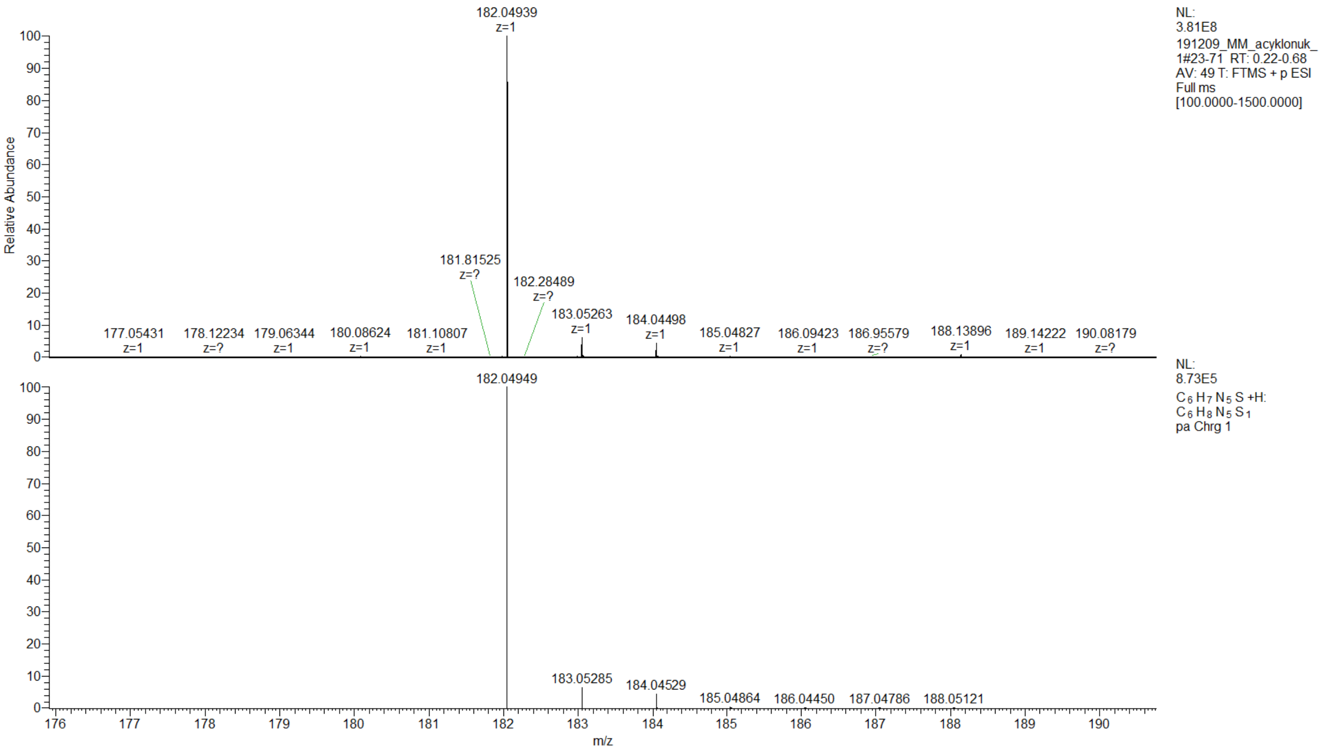

Compound 2

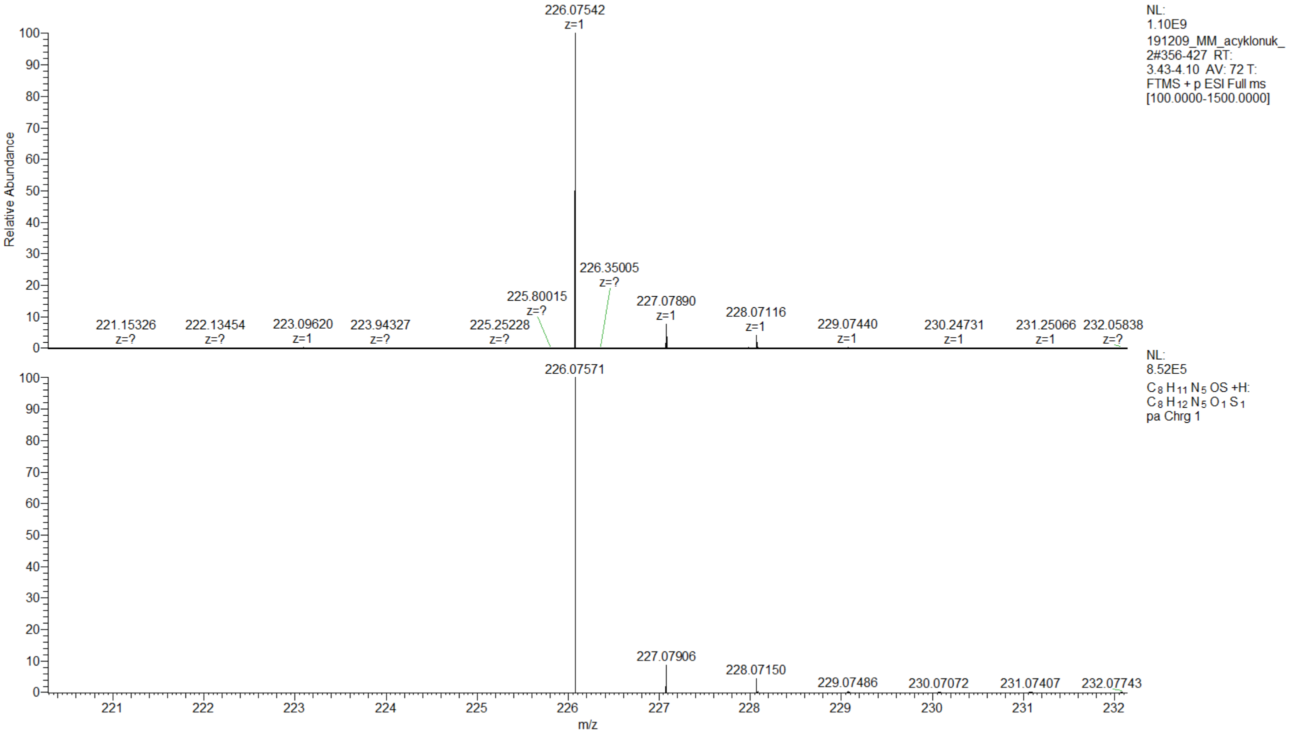

Compound 3

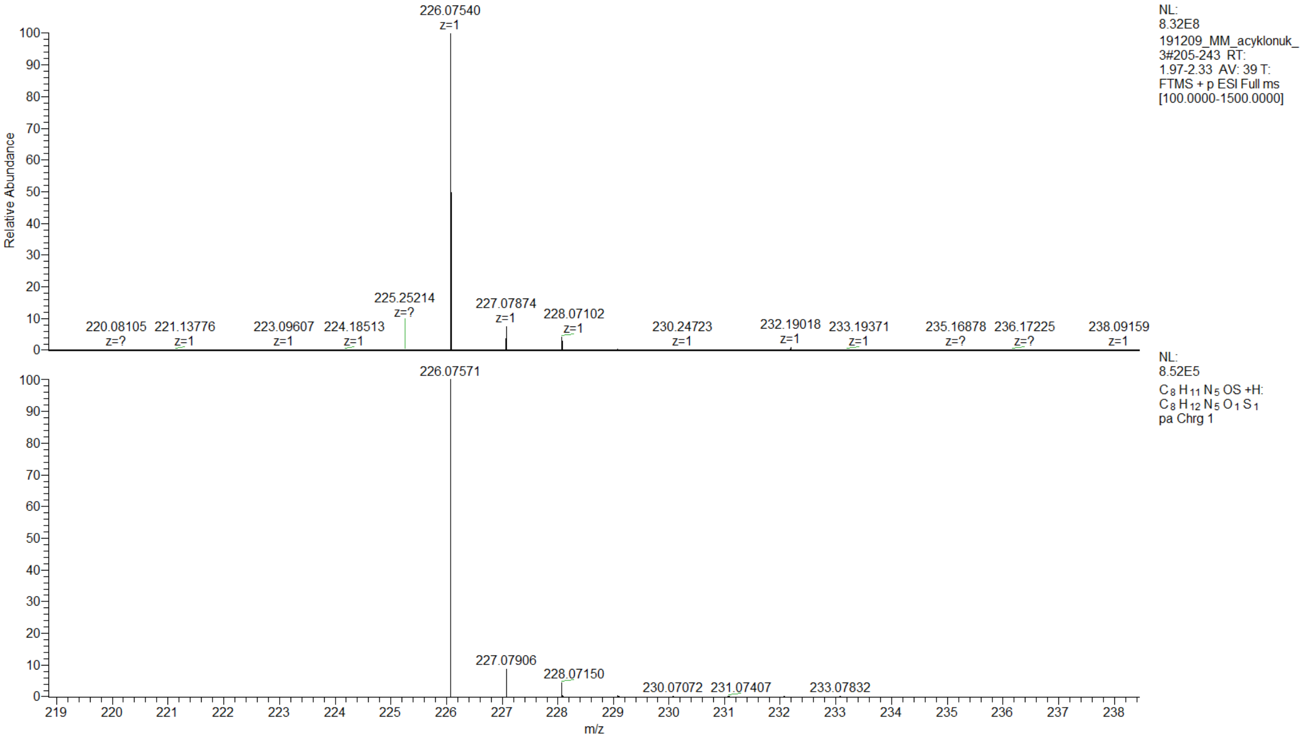

Compound 4

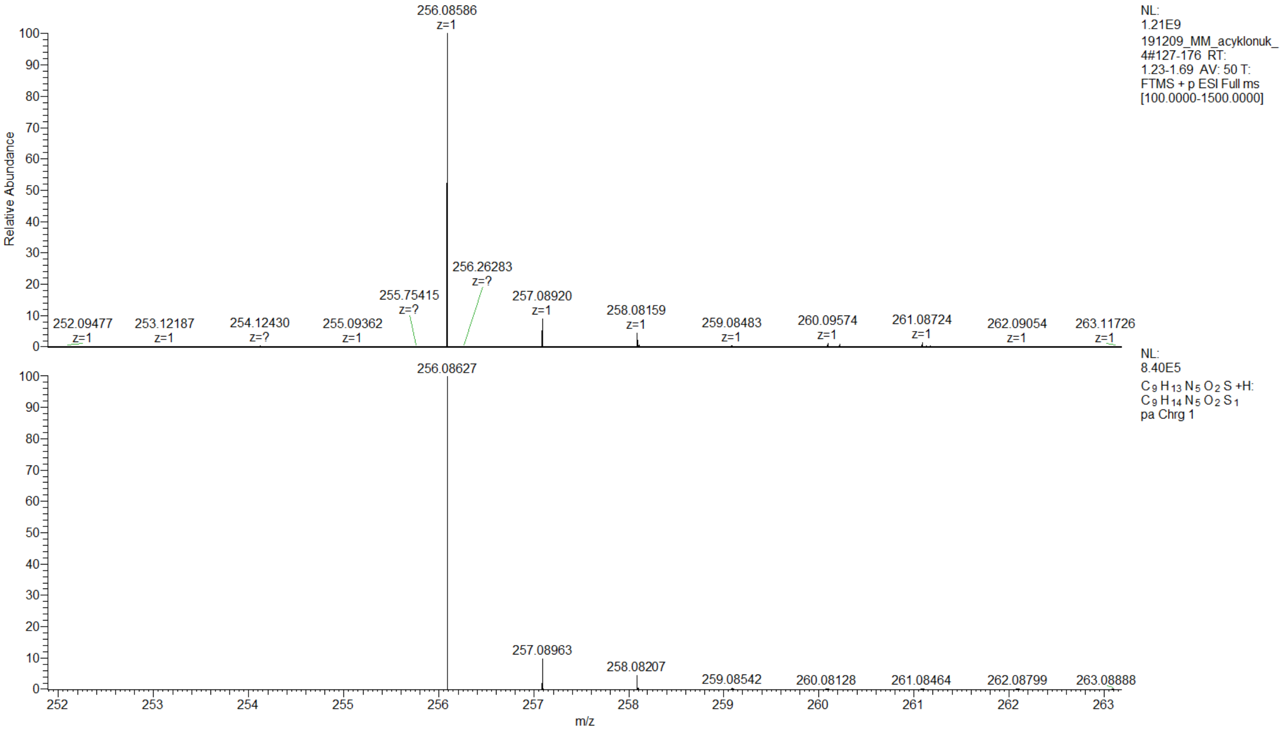

Compound 5

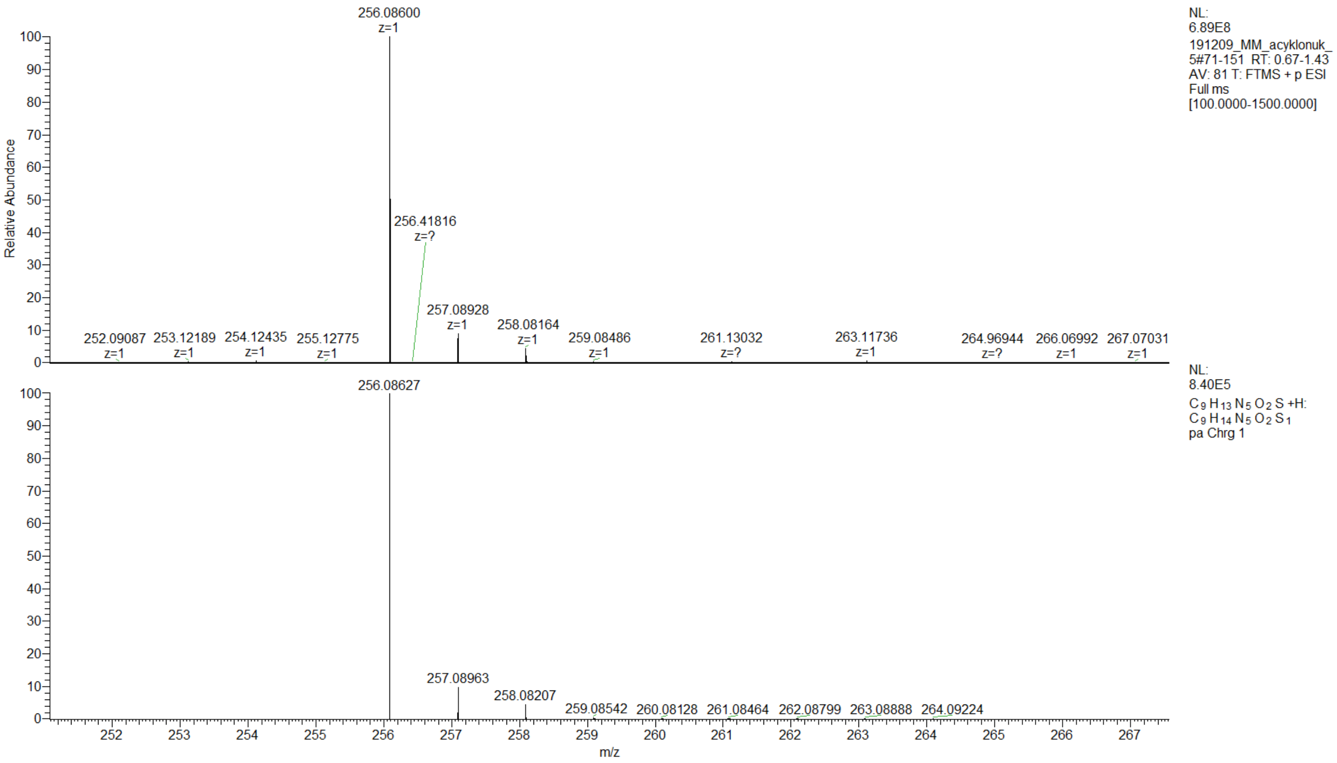

Compound 6a

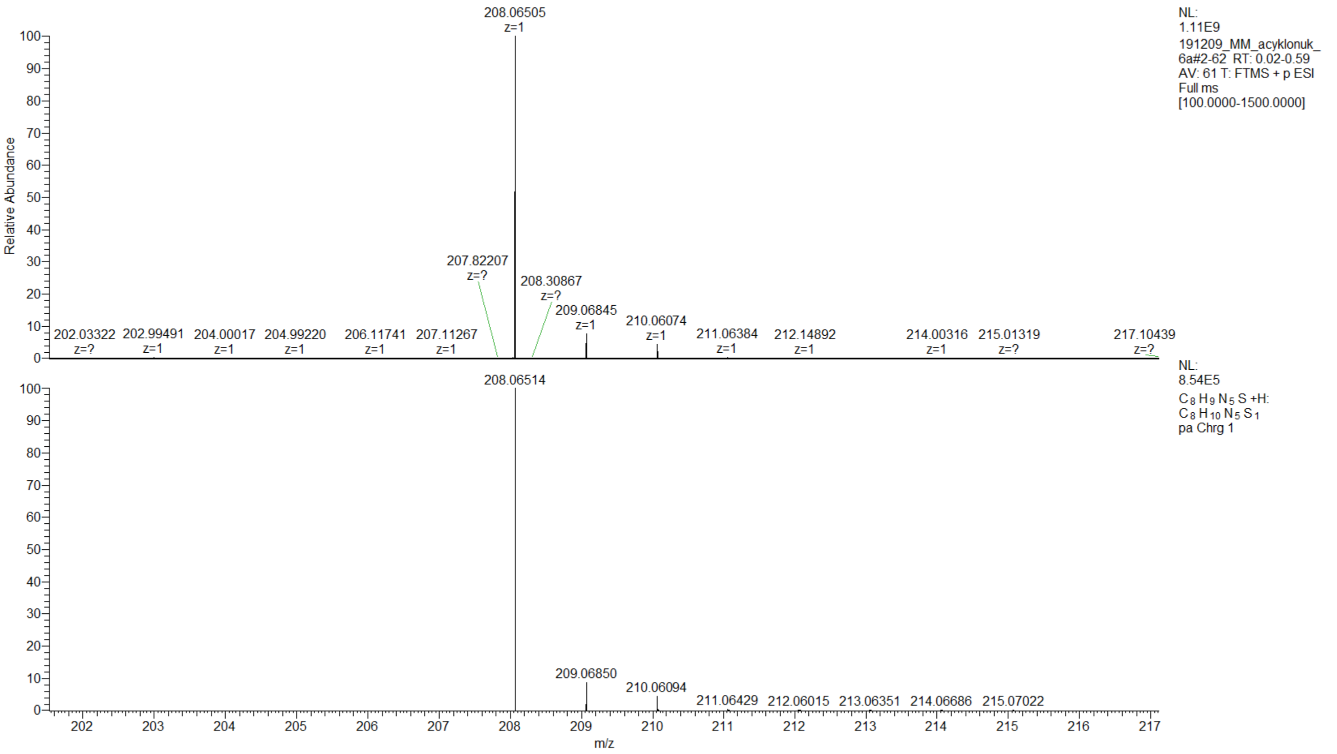

Compound 6

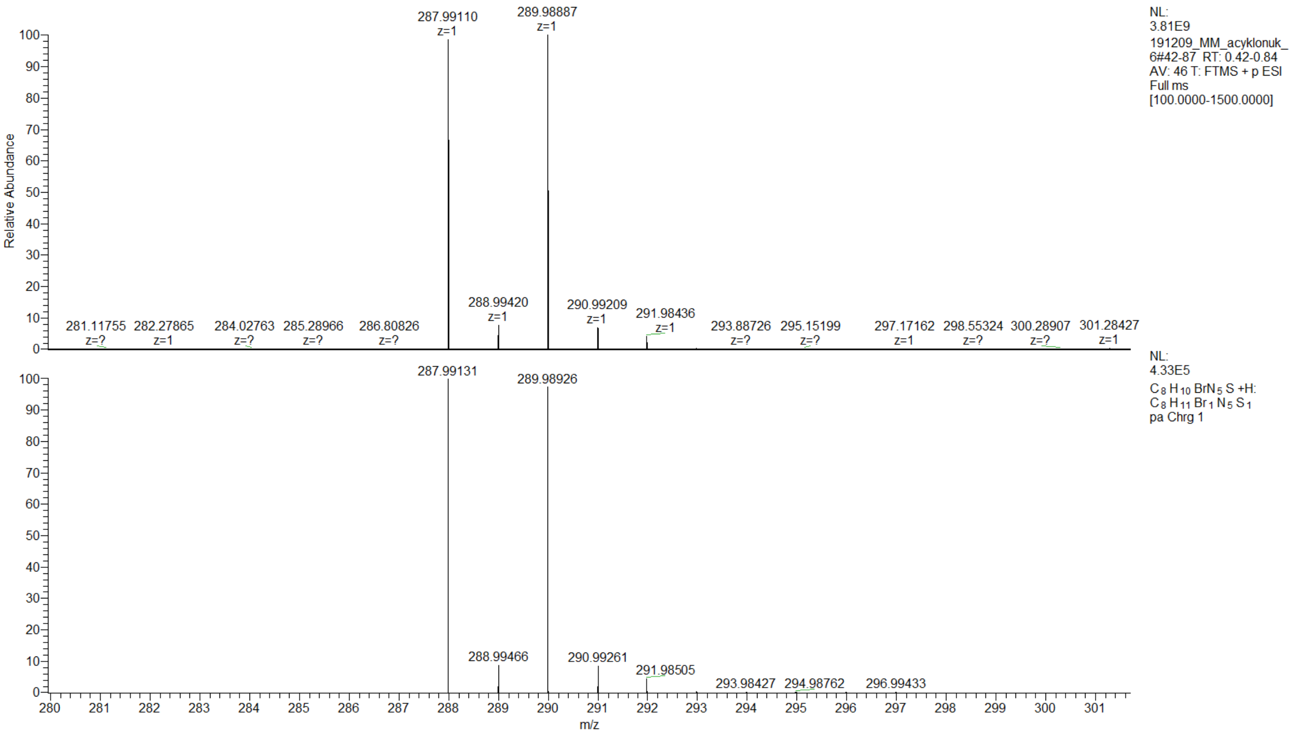

Compound 6b

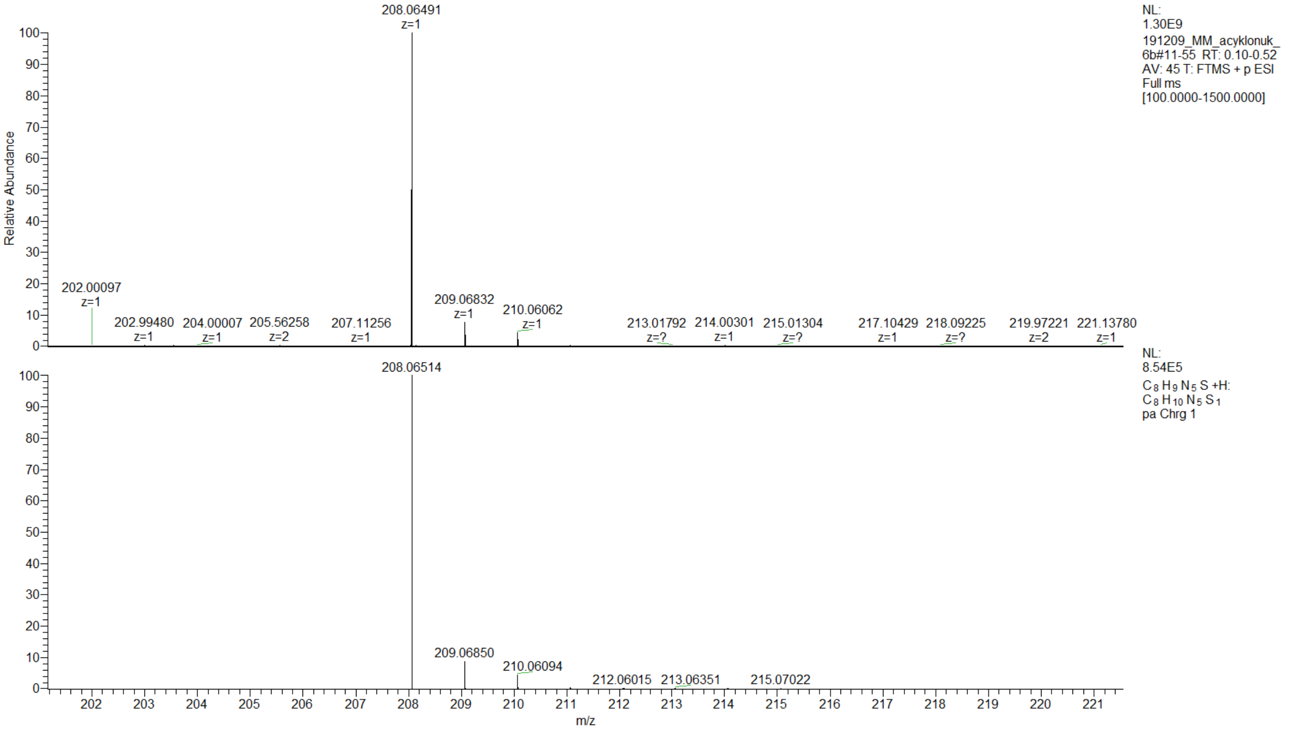

Compound 8

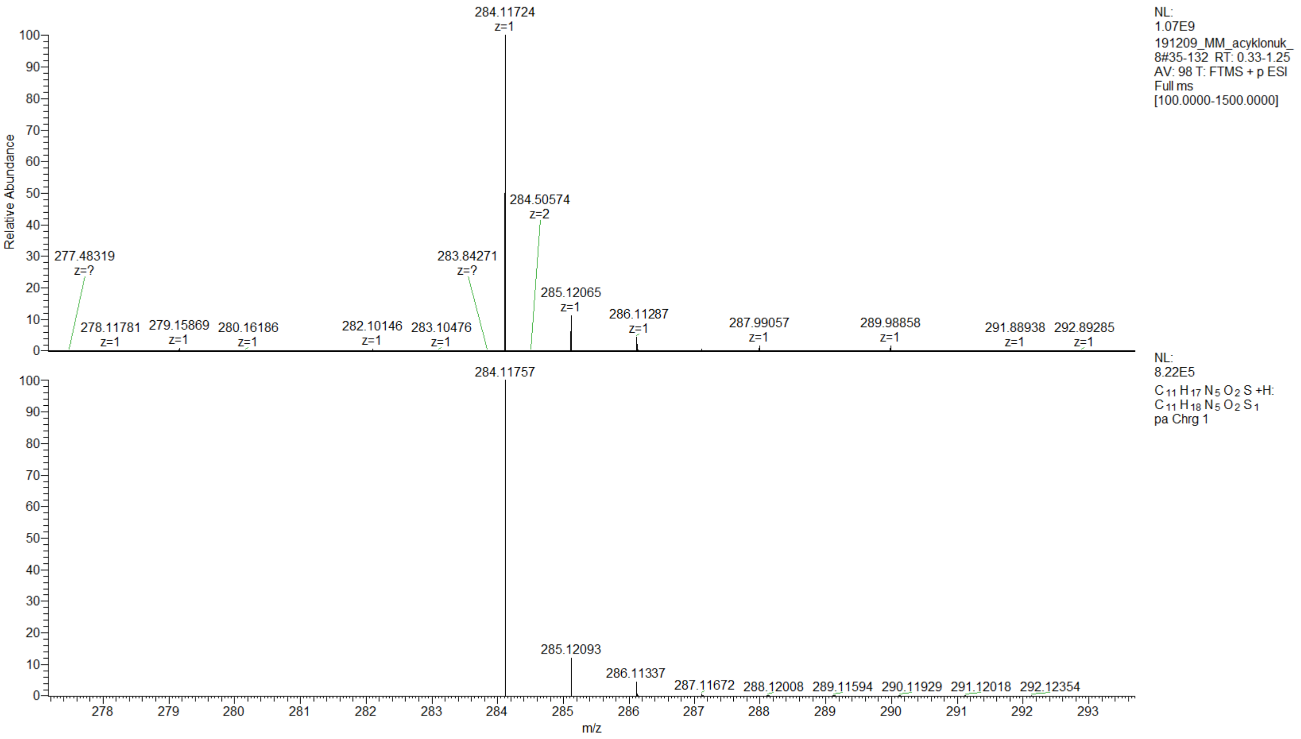

Compound 8a

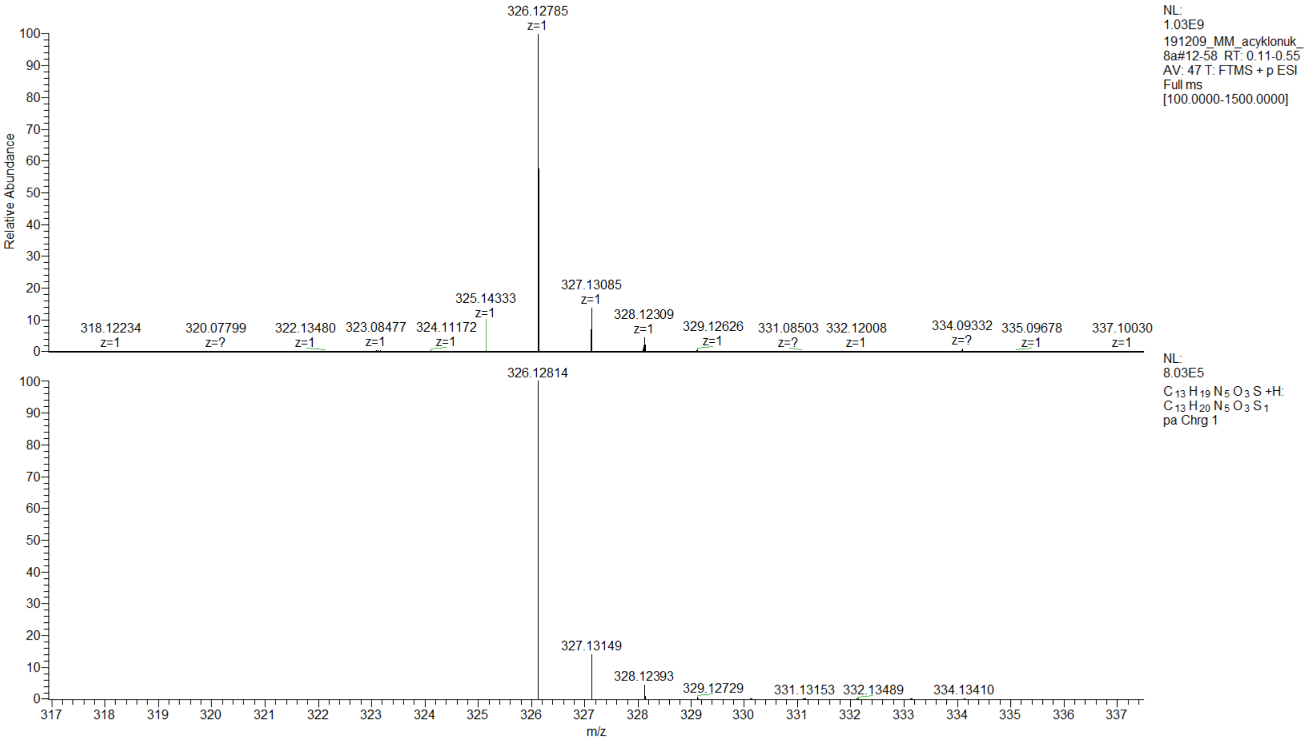

Coordinates for optimized geometries for molecules of compounds 1 – 8 obtained from DFT/B3LYP/6-311++G(d,p) calculations

Compound **1**

19
Molecule_Name_1

S	-2.99360	-0.18440	-0.00010
N	0.33840	-2.13320	-0.00020
N	-0.92180	-1.76360	-0.00000
N	-0.40210	0.59740	0.00030
N	2.58730	-1.19350	0.00000
N	3.12280	0.05420	0.00010
C	-1.24170	-0.43490	0.00030
C	0.88250	0.22510	0.00020
C	1.22830	-1.14210	-0.00010
C	2.12930	0.93030	-0.00010
C	2.37000	2.40250	-0.00020
C	-3.08220	1.63660	-0.00000
H	3.19010	-2.00060	-0.00020
H	3.44160	2.60320	-0.00420
H	1.92620	2.87110	0.88270
H	1.91910	2.87210	-0.87890
H	-4.14670	1.87340	-0.00010
H	-2.61090	2.04790	-0.89120
H	-2.61100	2.04790	0.89130

Compound **2**

26
Molecule_Name_2

S	3.68430	-1.18870	0.10860
N	-0.12720	-1.55860	-0.37910
N	1.16500	-1.75130	-0.24720
N	1.65190	0.59830	0.04700
N	-1.79480	0.21510	-0.40080
N	-1.76520	1.56610	-0.25550
C	1.99370	-0.68380	-0.04150
C	0.33560	0.79840	-0.08630
C	-0.53450	-0.29210	-0.29870
C	-0.50910	1.95050	-0.06950
C	-0.13620	3.38390	0.11300
H	-1.02950	4.00770	0.07590
H	0.55460	3.71090	-0.66920
H	0.36360	3.53920	1.07330
C	4.50250	0.41970	0.36410
H	5.56420	0.19180	0.46410

H	4.14020	0.90050	1.27140
H	4.34370	1.07600	-0.49010
C	-3.04940	-0.49530	-0.59020
C	-3.82170	-0.65400	0.71810
O	-5.02820	-1.33360	0.38580
H	-2.81220	-1.47560	-1.00500
H	-3.65210	0.05650	-1.31300
H	-4.02390	0.33490	1.14600
H	-3.22170	-1.23160	1.43250
H	-5.54330	-1.47220	1.18570

Compound **3**

26
Molecule_Name_3

S	3.98840	-0.02600	0.29080
O	-4.01010	0.91710	1.38830
H	-4.66800	1.56910	1.12990
N	0.82590	-2.19610	0.04120
N	2.02730	-1.73420	0.16890
N	1.38930	0.58890	-0.12460
N	-1.45520	-1.53760	-0.34120
N	-1.98870	-0.31490	-0.52740
C	2.26590	-0.36710	0.08180
C	0.12960	0.11240	-0.25760
C	-0.14990	-1.27840	-0.17420
C	-1.10430	0.72580	-0.48760
C	-3.43670	-0.24320	-0.70820
H	-3.71750	-1.11230	-1.30560
H	-3.67090	0.66080	-1.27330
C	-4.19630	-0.26880	0.62080
H	-5.25760	-0.44680	0.41210
H	-3.82640	-1.09840	1.22530
C	-1.44920	2.16560	-0.65120
H	-2.11090	2.51020	0.14720
H	-1.93180	2.36940	-1.61270
H	-0.52780	2.74800	-0.61030
C	3.99610	1.78840	0.12830
H	5.03720	2.08980	0.24830
H	3.38480	2.24820	0.90340
H	3.63360	2.08960	-0.85350

Compound **4**

30
Molecule_Name_4

S	4.18880	-1.34780	0.17750
N	0.43540	-1.42400	-0.72490
N	1.69590	-1.70930	-0.49220
N	2.27220	0.56210	0.09940

N	-1.12630	0.44280	-0.70540
N	-1.03900	1.76920	-0.43140
C	2.55160	-0.72410	-0.08650
C	0.98760	0.85580	-0.13390
C	0.08540	-0.15300	-0.53200
C	0.21110	2.05420	-0.09070
C	0.64210	3.43820	0.26320
H	-0.20710	4.11920	0.20200
H	1.42570	3.79030	-0.41380
H	1.04950	3.47280	1.27750
C	5.05410	0.16970	0.69730
H	6.08430	-0.13390	0.88620
H	4.61460	0.57440	1.60760
H	5.02800	0.92120	-0.09030
C	-2.40060	-0.17620	-1.02680
C	-3.37620	-0.13710	0.14710
C	-4.73170	-0.71480	-0.24310
H	-2.18160	-1.21020	-1.29920
H	-2.83050	0.33900	-1.88990
H	-3.51930	0.90780	0.45310
O	-2.79860	-0.88140	1.20930
O	-5.54140	-0.66260	0.93500
H	-5.18000	-0.12100	-1.04980
H	-4.60730	-1.75060	-0.58240
H	-3.46380	-0.94400	1.90550
H	-6.32230	-1.20980	0.81300

<u>Compound **5**</u>

30
Molecule_Name_5

S	4.68250	-0.29250	0.29710
O	-3.21110	1.24650	1.44620
H	-3.65640	2.02520	1.09210
N	1.33010	-2.15710	0.05690
N	2.56970	-1.80910	0.18370
N	2.15450	0.56030	-0.13580
N	-0.87830	-1.28960	-0.33680
N	-1.29390	-0.02440	-0.53550
C	2.93650	-0.47200	0.08380
C	0.85590	0.20320	-0.26760
C	0.44600	-1.15380	-0.17100
C	-0.31490	0.92770	-0.50490
C	-2.72760	0.18200	-0.71730
H	-3.08850	-0.60820	-1.37560
H	-2.88100	1.14500	-1.20890
C	-3.49310	0.12550	0.61110
C	-5.00550	-0.00880	0.38380
H	-3.13170	-0.74300	1.16840
C	-0.52060	2.39230	-0.68320

H	-1.12260	2.81640	0.12510
H	-1.00380	2.63090	-1.63600
H	0.45410	2.88170	-0.67160
C	4.86070	1.51210	0.12290
H	5.92500	1.71560	0.24450
H	4.29310	2.03210	0.89320
H	4.53050	1.83960	-0.86190
O	-5.35550	-1.14250	-0.39750
H	-5.37770	0.86010	-0.17110
H	-5.50190	-0.01610	1.36080
H	-5.22610	-1.94120	0.12500

Compound **6**

25
Molecule_Name_6

S	-4.52510	-1.56770	-0.13750
N	-0.72110	-1.37010	0.48360
N	-1.96670	-1.75110	0.31740
N	-2.77230	0.49440	-0.07580
N	0.67330	0.62640	0.49900
N	0.45690	1.95540	0.30200
C	-2.93170	-0.82080	0.04750
C	-1.50420	0.88490	0.09230
C	-0.49700	-0.06150	0.37140
C	-0.83240	2.14740	0.06130
C	-1.40150	3.50430	-0.18570
C	-5.55450	-0.10400	-0.48190
C	2.01360	0.11180	0.74110
C	2.81420	0.05400	-0.55700
Br	4.63820	-0.60690	-0.19950
H	-0.60890	4.25200	-0.15230
H	-2.15450	3.75490	0.56670
H	-1.89050	3.55060	-1.16270
H	-6.56750	-0.48660	-0.61070
H	-5.22870	0.39420	-1.39360
H	-5.52680	0.59570	0.35190
H	1.90560	-0.88260	1.17450
H	2.50170	0.76270	1.46700
H	2.92890	1.03970	-1.00040
H	2.37600	-0.63690	-1.27360

Compound **7**

46
Molecule_Name_7

S	6.01730	-1.61910	-0.16180
N	2.23500	-1.22880	-0.83020
N	3.47110	-1.65810	-0.71500
N	4.28940	0.43820	0.16880

N	0.86460	0.74720	-0.45890
N	1.08750	1.99820	0.02070
C	4.43680	-0.82310	-0.22700
C	3.03130	0.87840	0.05460
C	2.02170	0.02960	-0.44740
C	2.37190	2.11520	0.33310
C	2.94430	3.38030	0.87960
C	7.04960	-0.27990	0.51830
C	-0.47290	0.29990	-0.82100
C	-1.30210	-0.10290	0.40430
C	-2.68480	-0.64510	0.00820
H	3.39010	3.21380	1.86420
H	2.16120	4.13320	0.97150
H	3.73200	3.76790	0.22740
H	8.05550	-0.69560	0.58560
H	6.70320	0.01270	1.50820
H	7.04960	0.58450	-0.14370
H	-0.34240	-0.54370	-1.50250
H	-0.94720	1.11530	-1.37080
H	-1.41970	0.74720	1.07480
H	-0.77170	-0.88280	0.95680
C	-3.44300	-1.12030	1.26970
C	-3.55700	0.37650	-0.73320
O	-3.50940	1.58030	-0.15610
C	-4.33510	2.62770	-0.73980
C	-4.10790	3.88750	0.06790
O	-4.21470	0.11480	-1.71390
H	-5.37550	2.29740	-0.70940
H	-4.04900	2.74750	-1.78680
H	-4.39020	3.73880	1.11210
H	-4.71730	4.69730	-0.34190
H	-3.06020	4.19330	0.03150
O	-4.41060	-2.04910	1.13630
C	-4.68120	-2.79220	-0.08270
C	-3.83950	-4.05570	-0.15140
O	-3.21080	-0.66340	2.35620
H	-3.98800	-4.66600	0.74170
H	-4.13170	-4.64480	-1.02540
H	-2.77280	-3.83180	-0.23910
H	-5.74020	-3.03870	0.00050
H	-4.55700	-2.15470	-0.95750
H	-2.56810	-1.47880	-0.68510

<u>Compound **8**</u>

36
Molecule_Name_8

S	4.73600	-1.75370	0.35300
N	0.99980	-1.30220	-0.50700
N	2.20100	-1.76650	-0.24990

N	3.15390	0.42800	0.09810
N	-0.23510	0.78770	-0.68390
N	0.06930	2.10160	-0.52540
C	3.21720	-0.89890	0.03930
C	1.93080	0.90480	-0.16160
C	0.87000	0.02370	-0.46220
C	1.35630	2.21120	-0.21980
C	2.01000	3.53400	0.00350
H	1.27940	4.33450	-0.11590
H	2.82540	3.69270	-0.70780
H	2.43900	3.59440	1.00760
C	5.85140	-0.35620	0.70560
H	6.82190	-0.80650	0.91650
H	5.50770	0.20530	1.57290
H	5.92980	0.30570	-0.15540
C	-1.60110	0.35840	-0.95700
C	-2.48630	0.39760	0.29530
C	-3.94490	-0.01240	0.01680
H	-1.53040	-0.64900	-1.36870
H	-2.00110	1.01690	-1.73090
H	-2.45560	1.41640	0.69450
H	-2.05350	-0.25580	1.06180
C	-4.09160	-1.53700	-0.15160
C	-4.83410	0.51630	1.14350
O	-6.19110	0.11140	0.90290
O	-5.30260	-1.92310	-0.78140
H	-4.27820	0.45390	-0.91900
H	-3.98760	-2.01860	0.83410
H	-3.29170	-1.92900	-0.78360
H	-4.77190	1.61050	1.18640
H	-4.48780	0.11320	2.10530
H	-6.74010	0.36570	1.65030
H	-6.01970	-1.48730	-0.30170
